# Safety and Immunogenicity of an Additional Dose of Thailand Government Pharmaceutical Organization (GPO) Inactivated NDV-HXP-S COVID-19 Vaccine (HXP-GPOVac) Administered After Primary Vaccination with HXP-GPOVac or BNT162b2: An Open-Label Phase II Extension Trial in Thai Adults

**DOI:** 10.3390/vaccines14080660

**Published:** 2026-07-28

**Authors:** Prabda Praphasiri, Darunee Ditsungneon, Anusak Kerdsin, Sutthichai Nakphook, Jiraphut Kittiwatanachod, Kanlaya Sornwong, Suriya Naosri, Sarunpattori Khunarsa, Ponthip Wirachwong, Isariya Techatanawat, Piengthong Narakorn, Somchaiya Surichan, Jorge Flores, Laina D. Mercer, Christina S. Polyak, Bruce L. Innis, Rama Raghunandan, Chakrarat Pittayawonganon, Sopon Iamsirithaworn, Supakit Sirilak, Kriengkrai Prasert

**Affiliations:** 1 Influenza and Vaccine Study Center, Nakhon Phanom Provincial Hospital, Nakhon Phanom 48000, Thailand; 2 Faculty of Public Health, Kasetsart University Chalermphrakiat Sakon Nakhon Province Campus, Sakon Nakhon 47000, Thailand; 3 Government Pharmaceutical Organization (GPO), Bangkok 10400, Thailand; 4 PATH, Seattle, WA 98121, USA; 5 Office of the Permanent Secretary, Ministry of Public Health, Nonthaburi 11000, Thailand; 6 Bureau of Official Inspection, Office of the Permanent Secretary, Ministry of Public Health, Nonthaburi 11000, Thailand; 7 Health Systems Research Institute, Nonthaburi 11000, Thailand

**Keywords:** COVID-19 vaccine, phase II clinical trial, inactivated recombinant NDV-HXP-S vaccine, secondary immunization, Thailand

## Abstract

**Background/Objectives:** Waning immunity after primary COVID-19 vaccination supports evaluation of additional doses. HXP-GPOVac is an egg-based, inactivated Newcastle disease virus (NDV)-vectored vaccine expressing a prefusion-stabilized SARS-CoV-2 HexaPro spike antigen. We evaluated the safety, tolerability, and immunogenicity of a single additional 10 µg dose of HXP-GPOVac administered to adults previously primed with two doses of either HXP-GPOVac or BNT162b2. **Methods:** Study GPO NDV-HXP-S 203 was an open-label phase II extension enrolling adults (18–75 years) who previously completed a two-dose primary series in Study 202 with either HXP-GPOVac or BNT162b2 (Pfizer–BioNTech; Comirnaty). All participants received a single additional 10 µg intramuscular dose of HXP-GPOVac ≥ 6 months after their second primary dose. Solicited local/systemic adverse events (AEs) were recorded for 7 days, unsolicited AEs through Day 28, and serious AEs (SAEs) and adverse events of special interest (AESIs) throughout follow-up. Neutralizing antibody titers (pseudovirus 50% neutralization titer, NT_50_) and anti-spike IgG (BAU/mL) were assessed pre-dose (Day 1) and post-vaccination through 12 months; a predefined subset underwent IFN-γ and IL-5 ELISpot. SARS-CoV-2 infection during follow-up was assessed using anti-nucleocapsid (anti-N) IgG. Symptomatic COVID-19 was identified through symptom-reported, symptom-triggered RT-PCR testing; sequencing was performed when feasible. **Results:** All 219 participants received HXP-GPOVac (167 primed with HXP-GPOVac and 52 with BNT162b2). Any solicited local reaction occurred in 22.2% (37/167) of HXP-GPOVac-primed and 26.9% (14/52) of BNT162b2-primed participants; any solicited systemic reaction occurred in 10.8% (18/167) and 13.5% (7/52), respectively. No vaccine-related unsolicited AEs or AESIs were reported. Three deaths occurred during the 12-month follow-up; one (a sudden cardiac death in an HXP-GPOVac-primed participant) was assessed by the safety medical team as possibly related to vaccination, and two were assessed as not related. Neutralizing antibody GMTs increased from 46.33 at baseline to 1569.04 at Day 15 in HXP-GPOVac-primed participants and from 77.25 to 841.34 in BNT162b2-primed participants; corresponding SCRs were 78.8% and 76.9%. Anti-spike IgG GMCs increased from 48.79 to 1480.14 BAU/mL and from 194.48 to 1547.88 BAU/mL, respectively. Responses declined over time but remained above baseline through 12 months. In the cellular immunity subset, post-vaccination IFN-γ responses increased, with comparatively modest IL-5 responses and no pattern suggestive of Th2 predominance. **Conclusions:** A single additional dose of HXP-GPOVac administered ≥6 months after primary vaccination with HXP-GPOVac or BNT162b2 was generally well tolerated and elicited robust recall humoral responses, with supportive findings of cellular immunity. Trial registration: Thai Clinical Trials Registry, TCTR20230213001.

## 1. Introduction

Despite marked reductions in severe outcomes after vaccine rollout, SARS-CoV-2 continues to circulate globally and periodically cause substantial morbidity, mortality, and health-system disruption [1,2,3]. Ongoing transmission, viral evolution, and uneven vaccine access have sustained the need for vaccines that can be manufactured at scale with established infrastructure [1,2,4]. In Thailand, strengthening domestic vaccine production has been a long-standing preparedness priority, supported by investments in egg-based vaccine manufacturing capacity originally developed for influenza pandemic readiness and technology transfer initiatives [5,6,7]. These capabilities provide a practical foundation for locally produced COVID-19 vaccines and for rapid scale-up during future outbreaks [5,6,7].

HXP-GPOVac is an inactivated Newcastle disease virus (NDV)-vectored COVID-19 vaccine expressing a prefusion-stabilized SARS-CoV-2 spike antigen incorporating six proline substitutions (HexaPro, HXP) [8,9]. The NDV-HXP-S platform was developed to support high-yield egg-based production and has demonstrated robust neutralizing antibody responses and protection in preclinical models without evidence of vaccine-associated enhanced respiratory disease [10,11]. Early clinical development of NDV-HXP-S-based vaccines reported acceptable safety and immunogenicity profiles in Thailand and Vietnam, and a randomized phase 2 trial further supported immunogenicity in adults [12,13,14].

Waning humoral immunity after primary COVID-19 vaccination has been observed across platforms, motivating additional-dose strategies—particularly for older adults and those at increased risk of severe disease [15,16,17,18]. International and national guidance has evolved toward periodic revaccination for higher-priority groups [15,16,17], while also recognizing that heterologous and homologous additional-dose schedules can both enhance immune responses and clinical protection [19,20,21,22]. However, evidence gaps remain for additional dosing with egg-based NDV-HXP-S vaccines in populations primed with different platforms, and for the durability of responses after an additional dose in settings with ongoing SARS-CoV-2 exposure.

Study GPO NDV-HXP-S 203 (hereafter, Study 203) was designed as an open-label phase II extension of a prior randomized, double-blind phase II trial (Study 202) [23] to evaluate the safety, reactogenicity, and immunogenicity of a single additional 10 µg dose of HXP-GPOVac administered ≥6 months after completion of a two-dose primary series with either HXP-GPOVac or BNT162b2 (Pfizer–BioNTech [Pfizer Inc., New York, NY, USA; BioNTech SE, Mainz, Germany]; Comirnaty™). The extension also included predefined cellular immunity assessments (IFN-γ and IL-5 ELISpot) and surveillance for SARS-CoV-2 infection during follow-up using anti-nucleocapsid serology, with symptom-triggered RT-PCR testing and sequencing attempts for symptomatic cases.

## 2. Materials and Methods

### 2.1. Study Design, Objectives, and Outcomes

Study 203 was an open-label, non-randomized phase II extension enrolling adults aged 18–75 years who previously completed a two-dose primary series in Study 202 with either HXP-GPOVac or BNT162b2. The study was conducted at Nakhon Phanom Provincial Hospital and Nakae Hospital in Nakhon Phanom, Thailand. Participants were vaccinated between 15 April 2023 and 12 April 2024 and followed for 12 months after vaccination. Eligible participants received a single additional intramuscular dose of HXP-GPOVac on Day 1 of Study 203, administered ≥6 months after their second primary dose.

The primary objective was to assess the safety and tolerability of a single additional dose of HXP-GPOVac administered to adults previously primed with two doses of HXP-GPOVac or BNT162b2. Primary outcomes were the number and severity of solicited local and systemic adverse events (AEs) during Days 1–7, the number, severity, and relatedness of unsolicited AEs through Day 28, and the number of serious adverse events (SAEs) and adverse events of special interest (AESIs) through end of follow-up. Secondary objectives were to evaluate humoral immunogenicity by pseudovirus 50% neutralization titers (NT_50_) and anti-spike IgG, assess cell-mediated immunity in a predefined subset, and detect and characterize COVID-19 during follow-up. Secondary outcomes included NT_50_ geometric mean titers, anti-spike IgG geometric mean concentrations, geometric mean fold rises, and seroconversion rates (SCRs) at Day 15, Month 3, Month 6, and Month 12, as well as IFN-γ and IL-5 ELISpot responses in the cellular-immunity subset. Exploratory outcomes included vaccine-enhanced disease, anti-nucleocapsid serology as evidence of intercurrent SARS-CoV-2 exposure, and variant-specific neutralization analyses in a subset.

Primary safety outcomes were the number and severity of solicited local/systemic AEs during Days 1–7; the number, severity, and relatedness of unsolicited AEs through Day 28; and SAEs and AESIs through end of follow-up. Secondary immunogenicity outcomes included NT_50_ GMTs and anti-spike IgG GMCs at Day 15, Month 3, Month 6, and Month 12; geometric mean fold rises (GMFRs) from baseline; and seroconversion rates (SCRs). Cell-mediated immune outcomes included IFN-γ and IL-5 ELISpot responses and IFN-γ/IL-5 ratios in the predefined subset. Exploratory outcomes included intercurrent SARS-CoV-2 exposure detected by anti-N serology, symptomatic RT-PCR-confirmed COVID-19, and genetic sequencing results.

### 2.2. Sample Size

The sample size was justified separately for safety and immunogenicity. For safety, approximately 300 participants from Study 202 were expected to be available, including 225 HXP-GPOVac-primed and 75 BNT162b2-primed participants. The safety rationale focused primarily on the probability of observing at least one serious adverse event (SAE); among 200 homologous-boost participants, this probability was estimated to be 86.6% for a true SAE rate of 1.0% and 99.4% for a true SAE rate of 2.5%. For immunogenicity, which was a secondary descriptive objective rather than a formal between-group comparison, sample size was justified by the expected precision of 95% confidence intervals around geometric mean antibody estimates. The protocol anticipated that approximately 200 HXP-GPOVac-primed and 60 BNT162b2-primed participants would meet eligibility criteria for these descriptive immunogenicity analyses.

### 2.3. Vaccines and Vaccine Product

HXP-GPOVac is an egg-based, inactivated NDV-vectored vaccine expressing the prefusion-stabilized HexaPro spike antigen [8,9,10,11,12]. Each 0.5 mL intramuscular dose contained 10 µg of spike antigen without adjuvant, consistent with the formulation selected from earlier clinical development [12]. In the Study 202 primary series, participants had received two doses of either HXP-GPOVac (10 µg spike antigen per 0.5 mL dose) or BNT162b2 (30 µg mRNA per dose). The study vaccine was HXP-GPOVac, lot no. INP6506E (expiry date: 19 May 2023). The study vaccine was supplied as a multidose vial and stored at 2–8 °C with continuous temperature monitoring. Vaccine preparation followed sponsor instructions (gentle mixing; withdrawal of 0.5 mL; replacement with a 24G–26G needle prior to injection). Vaccination was administered into the deltoid muscle, preferably the non-dominant arm.

### 2.4. Enrollment, Randomization, and Blinding

The extension was open-label and non-randomized; all eligible participants received the same study vaccine (HXP-GPOVac) as the additional vaccination. Participants were required to be healthy or to have stable chronic medical conditions, be able to comply with study procedures, and not have received a third COVID-19 vaccine dose before enrollment.

Key exclusion criteria included receipt of an investigational medicinal product within 90 days, receipt of any non-study vaccine within 30 days before or after vaccination, history of anaphylaxis to immunizations, history of allergies to eggs/chicken or vaccine components, fever (≥38.0 °C) within 24 h, receipt of immunoglobulins or blood products within 3 months, continuous use of anticoagulants, clinically unstable or severe uncontrolled chronic disease, pregnancy or lactation, and current systemic immunosuppressive therapy. Participants with recent COVID-19 infection within 3 months or positive antigen testing at screening were excluded.

### 2.5. Safety Assessments

Participants were observed on-site for at least 30 min after vaccination for immediate reactions. Solicited local (pain/tenderness, erythema, swelling/induration) and systemic (fever defined as oral temperature ≥38 °C, headache, fatigue/malaise, myalgia, arthralgia, nausea/vomiting) adverse events (AEs) were recorded by participants on diary cards for 7 days after vaccination and reviewed at follow-up visits. Solicited reactions were captured for each day of this 7-day window; reported events were transient, with onset within the first days after vaccination and resolution within the solicited period. Events with onset within Days 1–7 that persisted beyond Day 7 continued to be recorded as solicited until resolution; events with onset after Day 7 were recorded as unsolicited AEs. Unsolicited AEs were collected through Day 28. Serious adverse events (SAEs) and adverse events of special interest (AESIs) were collected from Day 1 through the end of follow-up.

AEs were graded according to the Division of AIDS (DAIDS) Table for Grading the Severity of Adult and Pediatric Adverse Events, Version 2.1 (2017) [24] (Supplementary Table S6), and coded using MedDRA. Causality for unsolicited AEs, SAEs, and AESIs was assessed by investigators as related or not related; serious adverse events were additionally adjudicated by the safety medical team using a graded causality scale that included a possibly related category; causality was not assessed for solicited reactogenicity events.

### 2.6. Immunogenicity Assessments

Serum samples were collected pre-dose (Day 1) and at prespecified time points after vaccination (Day 15, Month 3, Month 6, and Month 12). Neutralizing antibody titers were measured using a validated pseudovirus neutralization assay and summarized as 50% neutralization titers (NT_50_) as geometric mean titers (GMTs) with 95% confidence intervals (CIs), as previously described [12]. Anti-spike IgG concentrations were measured by ELISA and summarized as geometric mean concentrations (GMCs) with 95% CIs, expressed in binding antibody units per milliliter (BAU/mL) based on assay calibration, as previously described [12]. Titers/concentrations below the lower limit of quantification (LLOQ) were imputed as LLOQ/2. Seroconversion was defined as a ≥4-fold increase from pre-dose baseline in NT_50_ or anti-spike IgG.

### 2.7. Cell-Mediated Immunity Assessment

A predefined subset contributed peripheral blood mononuclear cells (PBMCs) at baseline and prespecified post-vaccination time points (including approximately Day 8, Day 29, and Month 6). PBMCs were isolated, cryopreserved, and later stimulated with overlapping spike peptide pools. IFN-γ and IL-5 ELISpot assays were performed, and responses were expressed as spot-forming units (SFU) per 10^6^ PBMCs, with dimethyl sulfoxide (DMSO) and phytohemagglutinin (PHA) as negative and positive controls, respectively, using methods harmonized with prior Thai NDV-HXP-S studies.12 Ratios of IFN-γ to IL-5 were calculated descriptively as indices of Th1/Th2 polarization for the spike peptide pools.

### 2.8. Virological and Clinical Assessment

SARS-CoV-2 infection during follow-up was assessed using anti-nucleocapsid (anti-N) IgG measured as signal-to-cutoff (S/CO) ratios. Baseline anti-N status was defined using the Study 202 Month-6 (pre-additional-dose) stored serum specimen. Intercurrent SARS-CoV-2 exposure was defined as new anti-N IgG seropositivity (S/CO ≥ 1.1) after a baseline anti-N-negative specimen (manufacturer threshold). Participants with baseline anti-N seropositivity were considered previously infected and were excluded from analyses restricted to baseline anti-N-negative participants.

Symptomatic COVID-19 was identified through symptom-reported, symptom-triggered RT-PCR testing on respiratory specimens. Whole-genome sequencing was attempted on RT-PCR-positive specimens when quantity/quality permitted. Sequencing results are summarized in Supplementary Table S4.

### 2.9. Statistical Analysis

All analyses were prespecified in the Statistical Analysis Plan and performed using SAS^®^ version 9.4 (SAS Institute, Cary, NC, USA). All available data were included in listings and graphical summaries. Analyses were stratified by primary-series regimen (HXP-GPOVac-primed vs. BNT162b2-primed) and by prespecified age strata (18–59 years and 60–75 years).

Demographic and baseline characteristics were summarized by primary-series group and age stratum. Continuous variables were summarized as mean, standard deviation, minimum, median, and maximum; categorical variables were summarized as counts and percentages. Prior and concomitant medications were coded using the WHO Drug Dictionary, and medical history and AEs were coded using MedDRA. Participant disposition and reasons for withdrawal were summarized descriptively.

Safety analyses were performed in the safety population and summarized by primary-series group and age stratum. The percentages of participants with solicited and unsolicited AEs, SAEs, and AESIs were summarized with two-sided 95% confidence intervals calculated by the Clopper–Pearson exact method [25]. Solicited local and systemic AEs were summarized by severity, including immediate events within 30 min of vaccination and those recorded on diary cards during Days 1–7. Unsolicited AEs were summarized for events with onset during the first 28 days after vaccination and additionally summarized regardless of onset where relevant to COVID-19-associated events, with tabulations by maximum severity and relatedness as appropriate. AEs coded by MedDRA were summarized by system organ class and preferred term; summaries of preferred terms occurring in at least 2% of participants were produced, along with listings for SAEs and vaccination-related AEs.

Immunogenicity analyses were performed primarily in the per-protocol (PP) population, with intention-to-treat analyses as supportive. No adjustment for multiplicity was applied; all between-group and between-time-point comparisons are descriptive and should be interpreted accordingly. The PP population was defined as participants who received vaccination within allowable windows, had no major protocol deviations affecting immunogenicity, and did not experience breakthrough SARS-CoV-2 infection considered to impact immunogenicity assessments prior to the relevant time point. Participants seropositive for nucleocapsid antibody at baseline (anti-N ≥ 1.1) were excluded from seroresponse calculations in the PP analyses. NT_50_ titers were summarized as geometric mean titers (GMTs) and anti-spike IgG as geometric mean concentrations (GMCs), each with 95% confidence intervals at each time point. Geometric mean fold rises from baseline were calculated with 95% confidence intervals using log-transformed paired differences and back-transformation. The proportions of participants achieving seroconversion (≥4-fold increase from baseline) were presented with 95% confidence intervals based on the Clopper–Pearson method [25]. Reverse cumulative distribution curves were generated to describe response distributions. No imputation was performed for missing data; missing values were assumed to be missing at random, and all analyses used observed data at each time point.

Virology and clinical endpoints were summarized as the number of events and incidence rates (per 100 person-years). Intercurrent SARS-CoV-2 exposure was summarized using anti-N serology (anti-N ≥ 1.1) among participants who were anti-N seronegative in the pre-additional-dose baseline specimen (Study 202 Month-6). Symptomatic COVID-19 was summarized as symptom-reported, symptom-triggered RT-PCR-confirmed infection. Sequencing results, when available, were listed descriptively. Analyses addressing events potentially consistent with VED focused on respiratory-system adverse events and RT-PCR-confirmed COVID-19 cases.

Missing data were not imputed; analyses used available data at each time point.

### 2.10. Study Oversight

The study was sponsored by the Government Pharmaceutical Organization (GPO), conducted at clinical sites in Thailand, and performed in accordance with the Declaration of Helsinki and the International Council for Harmonisation guideline for Good Clinical Practice (ICH-GCP E6(R2)) [26]. The protocol and informed-consent documents were approved by the Ethical Review Committee for Research in Human Subjects, Ministry of Public Health, Thailand. All participants provided written informed consent before any study procedures. An independent Data and Safety Monitoring Board and protocol safety oversight processes reviewed accumulating safety data. The trial was registered with the Thai Clinical Trials Registry (TCTR20230213001; registered 13 February 2023).

## 3. Results

### 3.1. Study Enrollment and Baseline Characteristics

**Figure 1:**
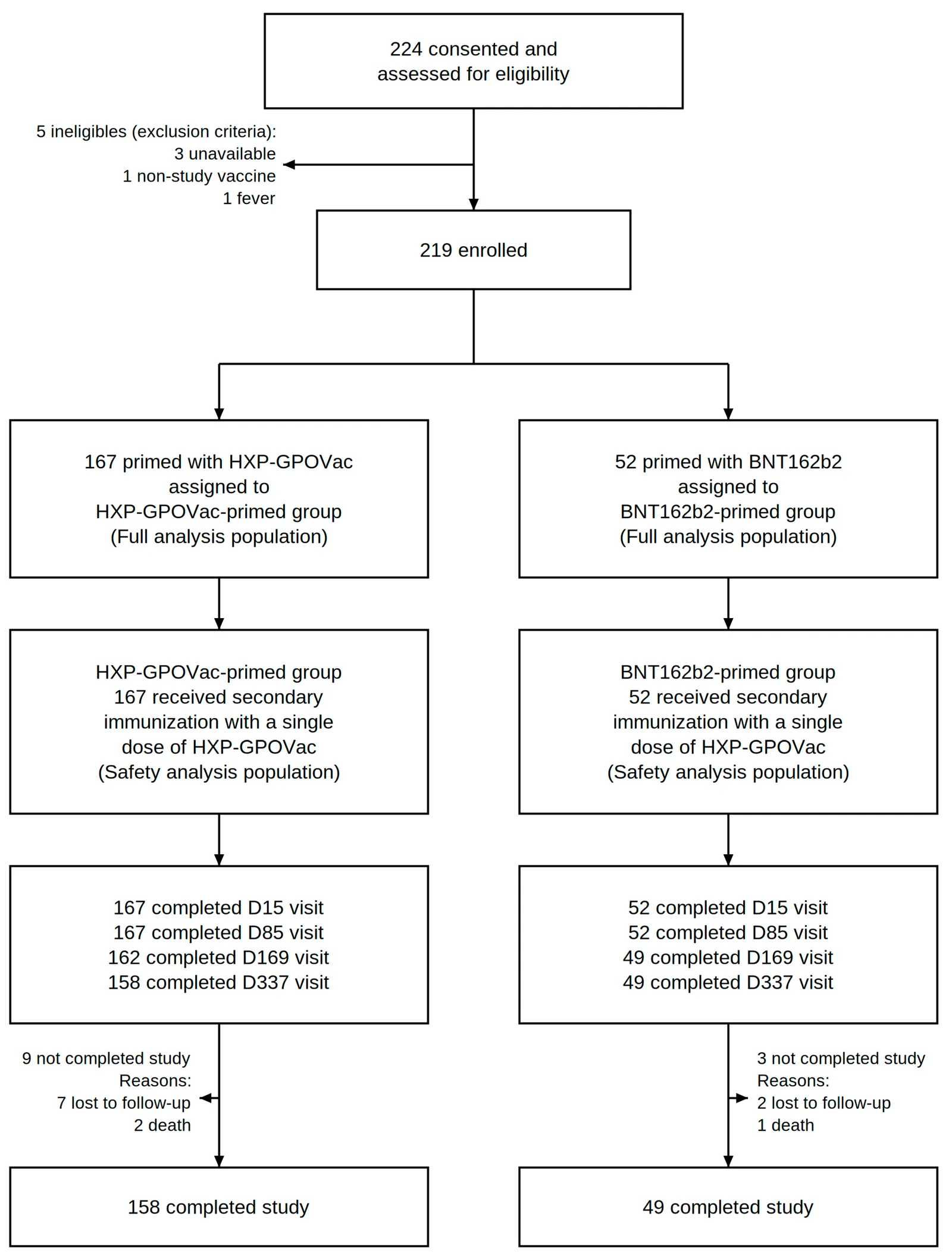
Disposition of study participants throughout the study.

Participant disposition is summarized in Figure 1 and Table 1. Enrollment began on 16 April 2023 and was completed on 23 April 2023. A total of 219 participants were enrolled and received the study vaccine (HXP-GPOVac) as an additional vaccination; 167 had received HXP-GPOVac, and 52 had received BNT162b2 as their primary series in Study 202. The overall mean age was 47.3 years (SD 13.6), with a median of 48.0 years (Q1–Q3 39.0–56.0) and a range of 18–74 years. Mean age was 48.4 (SD 13.1) in the HXP-GPOVac-primed group and 43.7 (SD 14.8) in the BNT162b2-primed group. Most participants were male (178/219, 81.3% overall; 137/167, 82.0% HXP-GPOVac-primed; 41/52, 78.8% BNT162b2-primed). Mean BMI was 23.0 kg/m^2^ (SD 3.8) overall (22.7, SD 3.6 in HXP-GPOVac-primed; 23.9, SD 4.2 in BNT162b2-primed). A total of 61 medical history events were recorded (NE = 61), including 45 events in HXP-GPOVac-primed and 16 in BNT162b2-primed; hypertension was the most frequently recorded preferred term (28/61 events, 45.9% overall; 20/45, 44.4% in HXP-GPOVac-primed; 8/16, 50.0% in BNT162b2-primed).

### 3.2. Safety and Reactogenicity

**Figure 2:**
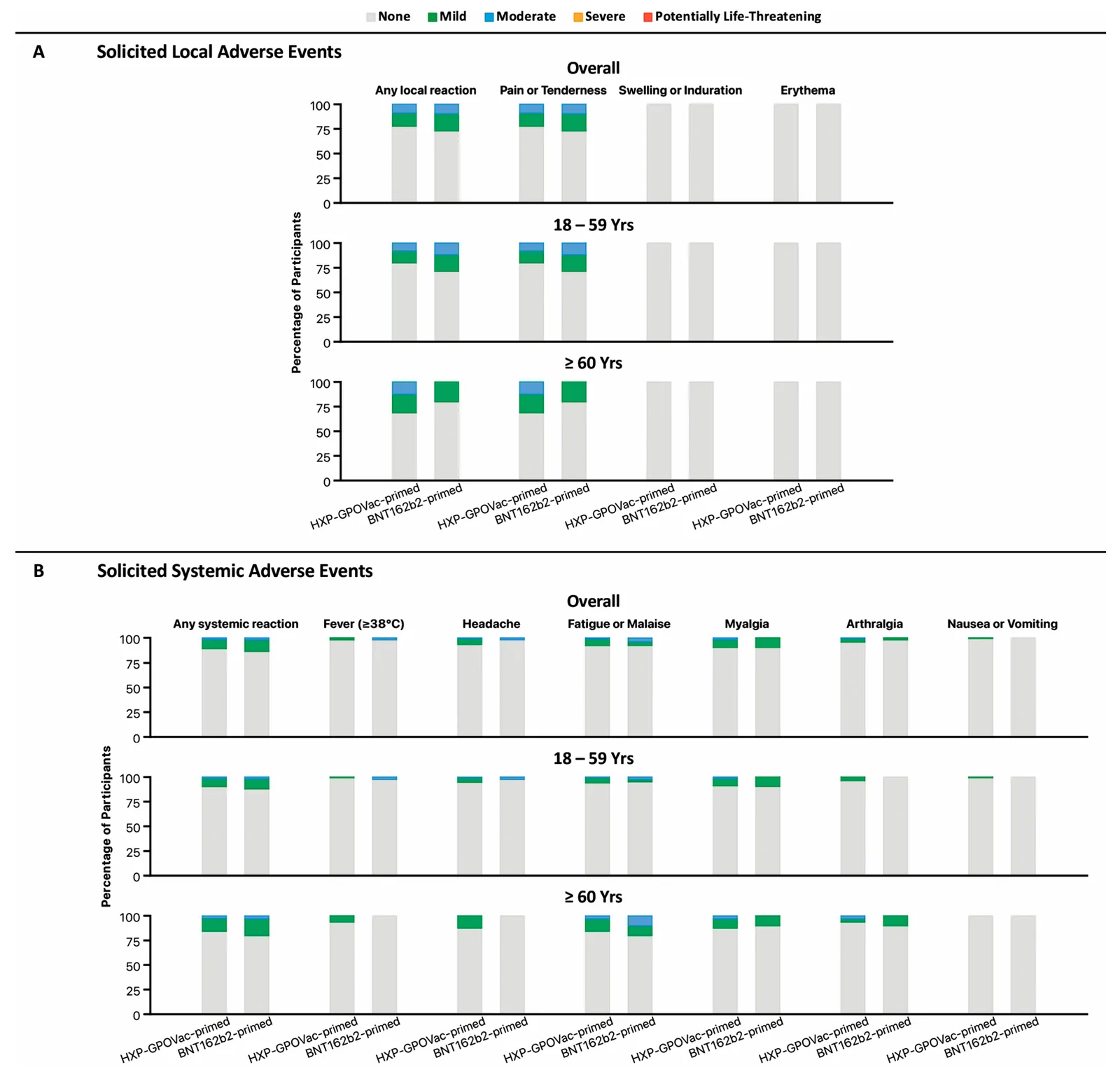
Summary of severity of solicited (**A**) local and (**B**) systemic adverse events during the first 7 days after vaccination by type and primary-series group, stratified by age (safety population).

Solicited local reactions within 7 days after vaccination are summarized in Figure 2 and Supplementary Table S1. Any solicited local reaction occurred in 22.2% of HXP-GPOVac-primed participants (37/167, 95% CI 16.1–29.2) and 26.9% of BNT162b2-primed participants (14/52, 95% CI 15.6–41.0). Injection-site pain or tenderness was the most common local event and occurred at the same frequency as “any local reaction” in both groups. Severity was predominantly mild to moderate: in the HXP-GPOVac-primed group, pain/tenderness was Grade 1 in 13.2% (22/167) and Grade 2 in 9.0% (15/167), with no Grade 3–4 events; in the BNT162b2-primed group, pain/tenderness was Grade 1 in 17.3% (9/52) and Grade 2 in 9.6% (5/52), with no Grade 3–4 events. Swelling/induration and erythema were not observed in either group (0% in both arms).

By age stratum, local reactogenicity was higher in ≥60 years than 18–59 years in the HXP-GPOVac-primed group (31.3% vs. 20.0%) and higher in 18–59 years than ≥60 years in the BNT162b2-primed group (28.6% vs. 20.0%).

Solicited systemic reactions within 7 days are summarized in Figure 2 and Supplementary Table S1. Any systemic reaction occurred in 10.8% with HXP-GPOVac-primed (18/167, 95% CI 6.5–16.5) and 13.5% with BNT162b2-primed (7/52, 95% CI 5.6–25.8). Fever (≥38 °C) was uncommon and occurred in 1.8% of HXP-GPOVac-primed participants (3/167, 95% CI 0.4–5.2) and 1.9% of BNT162b2-primed participants (1/52, 95% CI 0–10.3). Headache occurred in 6.6% with HXP-GPOVac-primed (11/167, 95% CI 3.3–11.5) and 1.9% with BNT162b2-primed (1/52, 95% CI 0–10.3). Fatigue/malaise occurred in 7.8% with HXP-GPOVac-primed (13/167, 95% CI 4.2–12.9) and 7.7% with BNT162b2-primed (4/52, 95% CI 2.1–18.5). Myalgia occurred in 9.6% with HXP-GPOVac-primed (16/167, 95% CI 5.6–15.1) and 9.6% with BNT162b2-primed (5/52, 95% CI 3.2–21.0). Arthralgia occurred in 4.2% with HXP-GPOVac-primed (7/167, 95% CI 1.7–8.4) and 1.9% with BNT162b2-primed (1/52, 95% CI 0–10.3). Nausea/vomiting was rare (0.6%, 1/167) with HXP-GPOVac-primed and was not reported with BNT162b2-primed. Across systemic symptoms, events were largely Grade 1 with small proportions Grade 2 and no Grade 3–4 for these systemic reactogenicity endpoints.

By age stratum, systemic reactogenicity was higher in ≥60 years than 18–59 years in both groups (HXP-GPOVac-primed 15.6% vs. 9.6%; BNT162b2-primed 20.0% vs. 11.9%).

Unsolicited adverse events within 28 days, SAEs, MAAEs, and AESIs are summarized in Table 2. Within 28 days after vaccination, any AE occurred in 4.2% with HXP-GPOVac-primed (7/167, 95% CI 1.7–8.4) and 3.8% with BNT162b2-primed (2/52, 95% CI 0.5–13.2). Most AEs were mild (Grade 1): 3.6% (6/167) with HXP-GPOVac-primed and 1.9% (1/52) with BNT162b2-primed; one moderate AE (Grade 2) occurred in the BNT162b2-primed group (1/52, 1.9%), and one severe AE (Grade 3) occurred in the HXP-GPOVac-primed group (1/167, 0.6%). No Grade 4–5 AEs occurred within 28 days in either group. No unsolicited AEs were determined to be vaccine-related in either group (0% with upper confidence bounds shown in Table 2).

Within 28 days, a SAE occurred in 0.6% of HXP-GPOVac-primed participants (1/167) and in 0% of BNT162b2-primed participants (0/52); none were vaccine-related, and none led to death within the 28-day window. MAAEs within 28 days occurred in 0.6% (1/167) with HXP-GPOVac-primed and 1.9% (1/52) with BNT162b2-primed; none were vaccine-related. No AESIs were reported in either group.

When summarized throughout the study, SAEs occurred in 8/167 (4.8%) in the HXP-GPOVac-primed group and 1/52 (1.9%) in the BNT162b2-primed group; three of these SAEs were fatal (two in HXP-GPOVac-primed participants and one in a BNT162b2-primed participant). One fatal SAE, a sudden cardiac death in an HXP-GPOVac-primed participant approximately 283 days after the additional dose, was assessed by the safety medical team as possibly related to vaccination following a recommendation from the ethics committee; the other two deaths (massive hemoptysis with acute respiratory failure secondary to pre-existing pulmonary tuberculosis, approximately 112 days; and a complication of cirrhosis, approximately 216 days) were assessed as not related. No AESIs were reported throughout follow-up.

### 3.3. Immunogenicity

**Figure 3:**
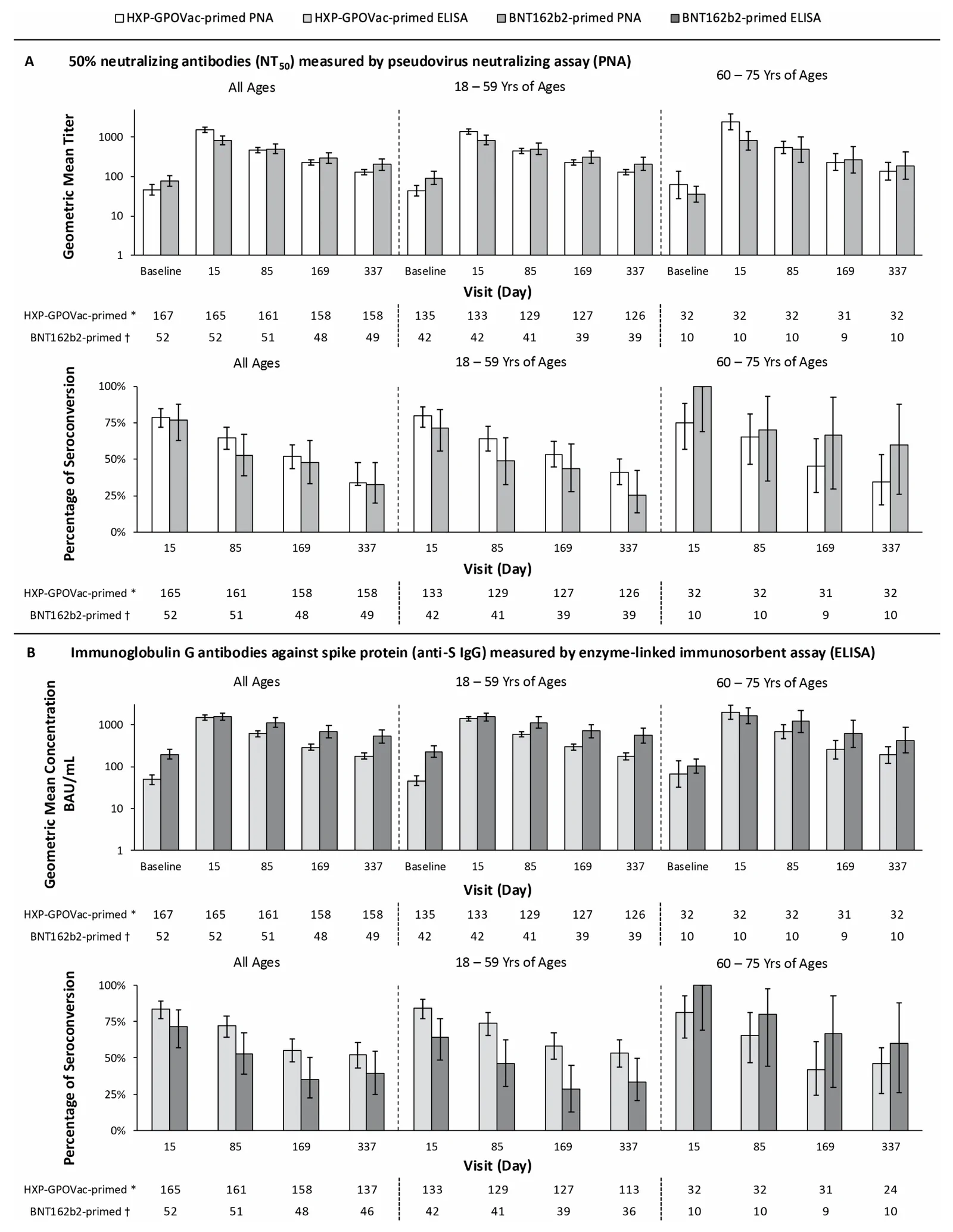
Geometric mean titers or concentrations and seroconversion rates of (**A**) pseudovirus neutralization NT_50_ and (**B**) anti-spike IgG at pre-dose baseline (Day 1) and post-vaccination time points (Day 15, Month 3 [Day 85], Month 6 [Day 169], and Month 12 [Day 337]) after an additional HXP-GPOVac dose, by primary-series group and age stratum (per-protocol population). **Abbreviations:** anti-spike IgG, anti–spike immunoglobulin G; BAU, binding antibody units; CI, confidence interval; ELISA, enzyme-linked immunosorbent assay; GMC, geometric mean concentration; GMT, geometric mean titer; NT_50_, 50% neutralization titer; PNA, pseudovirus neutralization assay. **Notes:** Numbers of participants with no missing data in both * HXP-GPOVac-primed and † BNT162b2-primed groups, at different time points, are shown in tables below the graphs. Error bars represent 95% CIs.

Neutralizing antibody titers (PNA NT_50_) and anti-spike IgG (ELISA) are summarized in Figure 3 and Supplementary Table S2, with between-group geometric mean ratios in Table 3.

At baseline (D1), NT_50_ GMT was 46.33 (95% CI 34.79–61.69) in the HXP-GPOVac-primed group (*n* = 167) and 77.25 (95% CI 55.80–106.94) in the BNT162b2-primed group (*n* = 52). At 14 days (D15), NT_50_ GMT increased to 1569.04 (95% CI 1329.51–1851.73) for HXP-GPOVac-primed (*n* = 165) and 841.34 (95% CI 659.86–1072.74) for BNT162b2-primed (*n* = 52), corresponding to GMFRs of 28.24-fold (95% CI 20.87–38.22) and 10.89-fold (95% CI 7.82–15.17), respectively, with SCRs of 78.8% (130/165; 95% CI 71.8–84.8) and 76.9% (40/52; 95% CI 63.2–87.5).

At 3 months (D85), NT_50_ GMT was 465.56 (95% CI 400.65–540.99) for HXP-GPOVac-primed (*n* = 161) and 506.93 (95% CI 376.72–682.15) for BNT162b2-primed (*n* = 51), with GMFRs of 8.42-fold (95% CI 6.34–11.19) and 6.59-fold (95% CI 4.26–10.22) and SCRs of 64.6% (104/161; 95% CI 56.7–72.0) and 52.9% (27/51; 95% CI 38.5–67.1).

At 6 months (D169), NT_50_ GMT was 230.15 (95% CI 195.20–271.37) for HXP-GPOVac-primed (*n* = 158) and 300.18 (95% CI 220.77–408.16) for BNT162b2-primed (*n* = 48), with GMFRs of 4.11-fold (95% CI 3.13–5.40) and 3.88-fold (95% CI 2.46–6.13) and SCRs of 51.9% (82/158; 95% CI 43.8–59.9) and 47.9% (23/48; 95% CI 33.3–62.8).

At 12 months (D337), NT_50_ GMT was 130.28 (95% CI 110.10–154.15) for HXP-GPOVac-primed (*n* = 158) and 205.06 (95% CI 145.95–288.10) for BNT162b2-primed (*n* = 49), with GMFRs of 2.44-fold (95% CI 1.87–3.20) and 2.87-fold (95% CI 1.76–4.69) and SCRs of 39.9% (63/158; 95% CI 32.2–48.0) and 32.7% (16/49; 95% CI 19.9–47.5).

The HXP-GPOVac-primed/BNT162b2-primed GMT ratio for NT_50_ was 0.60 (95% CI 0.35–1.03) at baseline, 1.86 (95% CI 1.35–2.58) at 14 days (D15), 0.92 (95% CI 0.67–1.26) at 3 months (D85), 0.77 (95% CI 0.54–1.08) at 6 months (D169), and 0.64 (95% CI 0.45–0.91) at 12 months (D337) (Table 3).

At baseline (D1), anti-S IgG GMC was 48.79 BAU/mL (95% CI 37.54–63.42) in the HXP-GPOVac-primed group (*n* = 167) and 194.48 BAU/mL (95% CI 148.26–255.10) in the BNT162b2-primed group (*n* = 52). At 14 days (D15), anti-S IgG GMC increased to 1480.14 (95% CI 1296.90–1689.27) for HXP-GPOVac-primed (*n* = 165) and 1547.88 (95% CI 1270.98–1885.10) for BNT162b2-primed (*n* = 52), corresponding to GMFRs of 28.11-fold (95% CI 21.66–36.49) and 7.96-fold (95% CI 6.01–10.54), respectively, with SCRs of 83.6% (138/165; 95% CI 77.1–88.9) and 71.2% (37/52; 95% CI 56.9–82.9).

At 3 months (D85), anti-S IgG GMC was 604.12 (95% CI 518.40–704.02) for HXP-GPOVac-primed (*n* = 161) and 1124.75 (95% CI 854.33–1480.76) for BNT162b2-primed (*n* = 51), with GMFRs of 11.54-fold (95% CI 8.87–15.00) and 5.74-fold (95% CI 3.96–8.32) and SCRs of 72.0% (116/161; 95% CI 64.4–78.8) and 52.9% (27/51; 95% CI 38.5–67.1). At 6 months (D169), anti-S IgG GMC was 286.81 (95% CI 242.05–339.85) for HXP-GPOVac-primed (*n* = 158) and 682.18 (95% CI 492.40–945.09) for BNT162b2-primed (*n* = 48), with GMFRs of 5.46-fold (95% CI 4.19–7.13) and 3.43-fold (95% CI 2.24–5.25) and SCRs of 55.1% (87/158; 95% CI 47.0–63.0) and 35.4% (17/48; 95% CI 22.2–50.5).

At 12 months (D337), anti-S IgG GMC was 177.80 (95% CI 149.86–210.93) for HXP-GPOVac-primed (*n* = 158) and 521.63 (95% CI 367.75–739.91) for BNT162b2-primed (*n* = 49), with GMFRs of 3.56-fold (95% CI 2.74–4.63) and 2.79-fold (95% CI 1.80–4.32). The seroconversion rate at 12 months (D337) was 51.8% (71/137; 95% CI 43.1–60.4) for HXP-GPOVac-primed and 39.1% (18/46; 95% CI 25.1–54.6) for BNT162b2-primed.

The HXP-GPOVac-primed/BNT162b2-primed GMC ratio for anti-S IgG was 0.25 (95% CI 0.15–0.41) at baseline, 0.96 (95% CI 0.74–1.24) at 14 days (D15), 0.54 (95% CI 0.39–0.73) at 3 months (D85), 0.42 (95% CI 0.30–0.60) at 6 months (D169), and 0.34 (95% CI 0.24–0.49) at 12 months (D337) (Table 3).

### 3.4. Cell-Mediated Immunity

**Figure 4:**
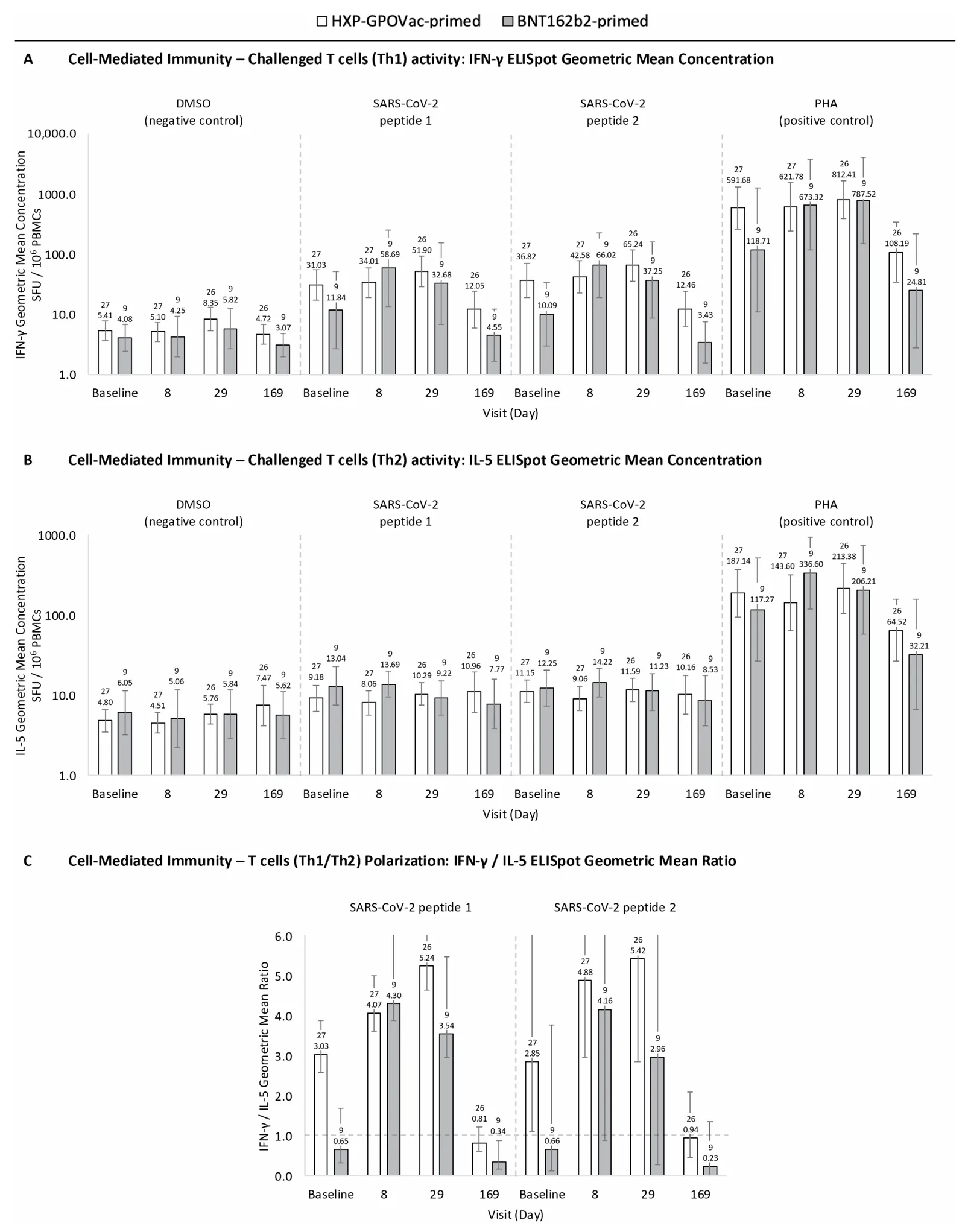
Cell-mediated immune responses to SARS-CoV-2 spike: (**A**) IFN-γ ELISpot GMCs, (**B**) IL-5 ELISpot GMCs, and (**C**) IFN-γ/IL-5 ELISpot GMRs at baseline (Day 1) and post-vaccination time points (Day 8, Day 29, and Month 6 [Day 169]) after an additional HXP-GPOVac dose, by primary-series group (per-protocol CMI subset). **Abbreviations:** CI, confidence interval; DMSO, dimethyl sulfoxide; ELISpot, enzyme-linked immunospot; GMC, geometric mean concentration; GMR, geometric mean ratio; IFN-γ, interferon gamma; IL-5, interleukin 5; PBMC, peripheral blood mononuclear cell; PHA, phytohemagglutinin; SFU, spot-forming units; Th1, T helper type 1; Th2, T helper type 2. **Notes:** IFN-γ and IL-5 ELISpot responses are expressed as SFU/10^6^ PBMCs. Above each bar, the upper value is the number of participants (*n*) and the lower value is the geometric mean concentration (**A**,**B**) or ratio (**C**). The dashed line in (**C**) marks a ratio of 1. Error bars represent 95% CIs.

Cell-mediated immune responses (CMI subset) are summarized in Figure 4 and Supplementary Table S3. At baseline (D1), spike-specific IFN-γ ELISpot responses to Peptide 1 were higher in the HXP-GPOVac-primed group (GMC 31.03 SFU/10^6^ PBMCs, 95% CI 17.50–55.01; *n* = 27) than in the BNT162b2-primed group (11.84, 95% CI 2.68–52.24; *n* = 9), while IL-5 responses were 9.18 (95% CI 6.30–13.39) and 13.04 (95% CI 7.51–22.64), respectively, yielding IFN-γ/IL-5 geometric mean ratios of 3.03 (95% CI 1.28–7.16) for HXP-GPOVac-primed and 0.65 (95% CI 0.11–3.75) for BNT162b2-primed. For Peptide 2 at baseline, IFN-γ GMCs were 36.82 (95% CI 18.94–71.57) and 10.09 (95% CI 2.99–34.04) for HXP-GPOVac-primed and BNT162b2-primed, respectively; IL-5 GMCs were 11.15 (95% CI 8.06–15.41) and 12.25 (95% CI 7.40–20.29), with IFN-γ/IL-5 ratios of 2.85 (95% CI 1.27–6.38) and 0.66 (95% CI 0.16–2.73).

At Day 8 (D8), IFN-γ responses increased for both peptide pools, with Peptide 1 IFN-γ GMC 34.01 (95% CI 19.11–60.53) in HXP-GPOVac-primed and 58.69 (95% CI 13.75–250.49) in BNT162b2-primed; IL-5 GMCs were 8.06 (95% CI 5.66–11.48) and 13.69 (95% CI 9.37–19.99), giving ratios 4.07 (95% CI 2.15–7.69) and 4.30 (95% CI 1.02–18.01), respectively. For Peptide 2 at D8, IFN-γ GMCs were 42.58 (95% CI 22.84–79.41) and 66.02 (95% CI 19.25–226.46), IL-5 GMCs were 9.06 (95% CI 6.36–12.90) and 14.22 (95% CI 9.38–21.56), and ratios were 4.88 (95% CI 2.60–9.15) and 4.16 (95% CI 1.05–16.53) for HXP-GPOVac-primed and BNT162b2-primed, respectively.

At Day 29 (D29), Peptide 1 IFN-γ GMC was 51.90 (95% CI 28.86–93.31) for HXP-GPOVac-primed and 32.68 (95% CI 6.80–156.96) for BNT162b2-primed; IL-5 GMCs were 10.29 (95% CI 7.46–14.21) and 9.22 (95% CI 5.61–15.14), with ratios 5.24 (95% CI 2.66–10.33) and 3.54 (95% CI 0.84–15.02). For Peptide 2 at D29, IFN-γ GMCs were 65.24 (95% CI 35.28–120.66) and 37.25 (95% CI 8.68–159.81), IL-5 GMCs were 11.59 (95% CI 8.25–16.28) and 11.23 (95% CI 6.76–18.66), and ratios were 5.42 (95% CI 2.45–11.97) and 2.96 (95% CI 0.63–13.88) for HXP-GPOVac-primed and BNT162b2-primed, respectively.

At Month 6 (D169), responses declined toward baseline. Peptide 1 IFN-γ GMC was 12.05 (95% CI 6.00–24.21) in HXP-GPOVac-primed and 4.55 (95% CI 1.68–12.33) in BNT162b2-primed, while IL-5 GMC was 10.96 (95% CI 6.13–19.59) and 7.77 (95% CI 3.83–15.76), yielding IFN-γ/IL-5 ratios of 0.81 (95% CI 0.33–1.96) and 0.34 (95% CI 0.08–1.45). For Peptide 2 at 6 months (D169), IFN-γ GMCs were 12.46 (95% CI 6.34–24.48) and 3.43 (95% CI 1.57–7.52), IL-5 GMCs were 10.16 (95% CI 5.81–17.76) and 8.53 (95% CI 4.16–17.49), and ratios were 0.94 (95% CI 0.44–1.98) and 0.23 (95% CI 0.08–0.64) for HXP-GPOVac-primed and BNT162b2-primed, respectively.

### 3.5. SARS-CoV-2 Infections During Follow-Up Identified by Anti-N Serology and Symptom-Triggered RT-PCR

Anti-nucleocapsid (anti-N) IgG results are summarized in Supplementary Table S5. For anti-N analyses, the pre-additional-dose “baseline” specimen was the stored serum collected at the Study 202 Month-6 post-primary-series visit; therefore, baseline anti-N denominators reflect participants who were anti-N IgG seronegative at that pre-additional-dose specimen (HXP-GPOVac-primed *n* = 144; BNT162b2-primed *n* = 48).

Among participants who were anti-N IgG seronegative at baseline (HXP-GPOVac-primed *n* = 144; BNT162b2-primed *n* = 48), anti-N IgG seropositivity (S/CO ≥ 1.1) was observed at Day 15 in 1/144 (0.7%) and 0/48 (0.0%), at Day 85 in 13/144 (9.0%) and 8/48 (16.7%), at Day 169 in 19/144 (13.2%) and 12/48 (25.0%), and at Day 337 in 32/144 (22.2%) and 16/48 (33.3%), respectively, consistent with intercurrent SARS-CoV-2 exposure during follow-up, including infections not necessarily captured through symptom-reported, symptom-triggered RT-PCR testing.

In the safety population, symptomatic RT-PCR–confirmed COVID-19 occurred in 5 participants overall (Supplementary Table S4), corresponding to an incidence density of 2.53 per 100 person-years; incidence densities were 1.87 per 100 person-years in participants aged 18–59 years (3 cases) and 5.33 per 100 person-years in participants aged 60–75 years (2 cases). Among symptomatic RT-PCR-confirmed COVID-19 cases with sequencing available, Omicron sublineages included XBB.1.16 (*n* = 2) and XBB.1.5.57 (*n* = 1); sequencing was unsuccessful in two cases.

## 4. Discussion

In this open-label phase II extension study, a single additional 10 µg dose of HXP-GPOVac administered ≥6 months after completion of a two-dose primary series with either HXP-GPOVac or BNT162b2 was generally well tolerated and elicited clear recall humoral responses, with supportive cellular immunity findings. The overall reactogenicity profile, predominantly mild to moderate solicited events, low frequency of unsolicited events, with no AESIs and one fatal serious adverse event (a sudden cardiac death) assessed as possibly related to vaccination, aligns with prior clinical experience for NDV-HXP-S-based vaccines in Thailand and Vietnam and with a randomized phase 2 evaluation of related formulations, which similarly reported acceptable safety profiles without evidence of clinically concerning immune-mediated safety signals [12,13,14].

Solicited local and systemic reactions were reported in a minority of participants, and severe reactogenicity was not observed for the solicited endpoints. Although cross-study comparisons should be interpreted cautiously, this pattern is consistent with expectations for inactivated protein-based or inactivated-virus platforms and with the NDV-HXP-S clinical development program [12,13,14]. The frequency and severity of solicited reactions were also lower than those commonly reported after mRNA-based additional doses in heterologous and homologous boosting studies such as COV-BOOST and the mix-and-match evaluation by Atmar et al., in which systemic events including fatigue, myalgia, and fever were more frequent; this difference is consistent with the generally milder reactogenicity of inactivated and protein-based platforms and may favor acceptability in booster programs [19,20]. In addition, no AESIs were identified. While the study was not designed to detect rare adverse events, the absence of AESIs in this extension is directionally consistent with preclinical NDV-HXP-S studies that demonstrated protective immunity without evidence of vaccine-associated enhanced respiratory disease (VAERD)-type pathology [10,11,27].

Three deaths occurred during the 12-month follow-up, all in participants who had received the additional HXP-GPOVac dose. Two were assessed as not related to vaccination: a death from massive hemoptysis with acute respiratory failure on a background of previously treated pulmonary tuberculosis approximately 112 days after dosing, and a death from complications of cirrhosis approximately 216 days after dosing. The third, a sudden cardiac death in a 42-year-old HXP-GPOVac-primed man approximately 283 days after the additional dose, was assessed by the safety medical team, following a recommendation from the ethics committee, as possibly related to the study vaccine. No adverse events of special interest were reported during follow-up. Given the open-label, single-arm design and the limited sample size, continued pharmacovigilance in larger and more diverse populations is warranted to further characterize the safety profile of the additional dose.

Neutralizing antibody titers (NT_50_) and anti-spike IgG concentrations increased substantially by Day 15 after the additional dose in both primary-series groups, consistent with an anamnestic response. Several features of the response are best interpreted through the lens of immune history. Participants primed with BNT162b2 entered the extension with higher baseline anti-spike IgG than those primed with HXP-GPOVac, whereas post-dose peak neutralization was higher in the HXP-GPOVac-primed group. Because the extension was non-randomized and groups differed in baseline antibody levels and potentially in intercurrent exposure risks, these between-group contrasts should be treated as descriptive rather than causal. Nonetheless, the findings are consistent with the broader evidence that post-dose magnitude is strongly influenced by baseline titers, time since primary series, and heterologous versus homologous histories [19,20,21,22]. Our finding that a single HXP-GPOVac additional dose elicited clear recall responses in both homologous (HXP-GPOVac-primed) and heterologous (BNT162b2-primed) recipients is concordant with mix-and-match booster trials reporting that both schedules are immunogenic and that the magnitude of the post-dose response is shaped by the priming platform, the interval since the primary series, and baseline immunity; the higher post-dose neutralization observed in the HXP-GPOVac-primed group, despite lower baseline binding antibody, should nonetheless be interpreted cautiously in light of the non-randomized design [19,20].

Neutralizing antibodies and binding anti-spike IgG are informative quantitative markers because higher levels have been associated with lower risk of symptomatic infection in correlates and immunobridging analyses across multiple COVID-19 vaccine programs; however, these relationships are continuous and variant-dependent, and no single protective threshold can be assumed across assays or platforms [28,29]. Therefore, the strong post-dose increases observed here support immunologic boosting, but they should not be over-interpreted as direct evidence of comparative effectiveness, particularly because the assays are based on ancestral antigens and because circulating variants during follow-up included Omicron sublineages.

In the predefined cellular immunity subset, post-vaccination IFN-γ responses increased with comparatively modest IL-5 responses, and there was no pattern suggesting Th2 predominance. Although the subset was small (especially in the BNT162b2-primed group) and ELISpot measures are inherently variable, these descriptive findings are directionally consistent with prior NDV-HXP-S clinical and preclinical work indicating induction of spike-responsive cellular immunity without signals suggestive of Th2-skewed immunopathology [10,11,12,13,14,27]. Given historical concerns about Th2-biased responses and enhanced respiratory disease for some respiratory virus vaccine approaches, these cellular data are supportive, but they are not definitive [27]. In this respect, the Th1-oriented profile observed here, with IFN-γ responses exceeding IL-5, resembles the cell-mediated responses reported for mRNA and adjuvanted protein-subunit COVID-19 vaccines, and differs from the Th2-skewed responses historically associated with certain formalin-inactivated respiratory-virus vaccines that raised enhanced-disease concerns.

Both neutralizing titers and anti-spike IgG waned after the early post-dose peak but remained above baseline through 12 months. Waning after peak is expected across COVID-19 vaccine platforms, including mRNA vaccines, and is commonly observed within the first months after vaccination before reaching a lower plateau [30]. More detailed cross-platform comparison is instructive: the decline observed here after the additional dose broadly parallels the waning kinetics reported for mRNA and protein-based COVID-19 boosters, in which neutralizing titers fall most steeply during the first two to three months before stabilizing at a lower plateau rather than returning to pre-boost levels. Direct durability comparisons across studies nonetheless remain limited by differences in assays, sampling schedules, and the variants circulating during follow-up, and residual titers cannot be equated with a defined level of protection [28,29,30]. A key interpretive limitation is antigenic evolution: immune assays based on Wuhan-like spike remain valuable for internal consistency and comparison to earlier NDV-HXP-S studies, but they do not directly quantify neutralization of later Omicron sublineages. Consequently, variant-relevant neutralization and real-world protection cannot be inferred from ancestral-strain titers alone [28,29].

This extension used two complementary approaches to identify intercurrent SARS-CoV-2 exposure and symptomatic infection during follow-up: anti-nucleocapsid (anti-N) serology to detect infections not necessarily captured through symptom-triggered testing, and symptom-reported, symptom-triggered RT-PCR to identify symptomatic RT-PCR–confirmed COVID-19. Both approaches have limitations in vaccinated cohorts. Anti-N seroconversion may be absent after post-vaccination infections, and anti-N responses can wane over time, which can under-ascertain prior infection when anti-N is used as a primary marker of intercurrent exposure [31,32]. Symptom-triggered RT-PCR under-ascertains infections not reported at scheduled contacts and is sensitive to symptom recall and reporting. Accordingly, anti-N serology is interpreted as evidence of intercurrent exposure rather than a definitive classification of asymptomatic infection.

This study has several limitations typical of extension designs. The open-label, non-randomized structure increases susceptibility to confounding by immune history, baseline titers, time since primary series, and intercurrent exposure. The study population was predominantly male, which may limit the generalizability of reactogenicity and safety estimates to more sex-balanced populations. However, sex-stratified safety analyses were not prespecified, and this study was not designed to determine whether sex composition materially affected AE reporting. The sample size supports characterization of common reactogenicity events but is underpowered for rare adverse events. Assays were based on ancestral antigens, limiting direct extrapolation to currently circulating variants. The cellular immunity subset was modest, restricting precision for Th1/Th2 characterization. Finally, infection ascertainment methods (anti-N and symptom-triggered RT-PCR) likely under-captured the full spectrum of infections during follow-up [31,32].

## 5. Conclusions

A single additional dose of HXP-GPOVac administered ≥6 months after primary vaccination with either HXP-GPOVac or BNT162b2 was generally well tolerated and elicited robust recall humoral responses with supportive cellular immunity findings. The results extend prior NDV-HXP-S clinical experience and support further evaluation focused on durability, baseline-adjusted comparisons across immune histories, and variant-relevant immunogenicity endpoints, ideally in larger and more sex-balanced cohorts with longer follow-up [10,11,12,13,14,19,20,21,22,28,29,30].

## Figures and Tables

**Table 1:** Baseline characteristics of study participants by primary-series group (safety population).

Characteristics	All (*N* = 219)	Vaccine Group	
		HXP-GPOVac-Primed	BNT162b2-Primed
		(*N* = 167)	(*N* = 52)
Age—yrs			
Mean (SD)	47.3 (13.6)	48.4 (13.1)	43.7 (14.8)
Median (Q1–Q3)	48.0 (39.0–56.0)	49.0 (40.0–57.0)	45.5 (34.5–52.0)
Min, Max	18, 74	19, 74	18, 71
Sex—*n* (%)			
Male	178 (81.3)	137 (82.0)	41 (78.8)
Female	41 (18.7)	30 (18.0)	11 (21.2)
Height—cm			
Mean (SD)	160.6 (7.6)	160.5 (7.7)	160.8 (7.3)
Median (Q1–Q3)	161.0 (155.0–166.0)	161.0 (155.0–166.0)	161.0 (155.5–166.0)
Min, Max	137, 181	137, 181	143, 175
Weight—kg			
Mean (SD)	59.2 (10.5)	58.5 (10.3)	61.6 (10.7)
Median (Q1–Q3)	58.0 (52.0–65.0)	57.0 (51.0–64.0)	60.0 (54.5–67.0)
Min, Max	36, 96	36, 96	36, 92
Body Mass Index (BMI)—kg/m^2^			
Mean (SD)	23.0 (3.8)	22.7 (3.6)	23.9 (4.2)
Median (Q1–Q3)	22.6 (20.0–24.5)	22.3 (19.8–24.4)	23.1 (21.8–26.5)
Min, Max	14.4, 34.0	16.0, 32.4	14.4, 34.0
Medical History by System Organ Class (SOC) and Preferred Term (PT)—*NE* (%)			
NE	61	45	16
All SOC			
All PT—NE (%)	61 (100)	45 (100)	16 (100)
Cardiac disorders			
All	3 (4.9)	2 (4.4)	1 (6.3)
Acute myocardial infarction	1 (1.6)	0 (0)	1 (6.3)
Arrhythmia	1 (1.6)	1 (2.2)	0 (0)
Coronary artery disease	1 (1.6)	1 (2.2)	0 (0)
Infections and infestations			
Tuberculosis	1 (1.6)	1 (2.2)	0 (0)
Metabolism and nutrition disorders			
All	12 (19.7)	7 (15.6)	5 (31.3)
Diabetes mellitus	8 (13.1)	5 (11.1)	3 (18.8)
Dyslipidemia	4 (6.6)	2 (4.4)	2 (12.5)
Musculoskeletal and connective tissue disorders			
Gouty arthritis	1 (1.6)	1 (2.2)	0 (0)
Nervous system disorders			
All	5 (8.2)	4 (8.9)	1 (6.3)
Cerebrovascular accident	2 (3.3)	1 (2.2)	1 (6.3)
Epilepsy	2 (3.3)	2 (4.4)	0 (0)
Post-traumatic epilepsy	1 (1.6)	1 (2.2)	0 (0)
Psychiatric disorders			
All	4 (6.6)	4 (8.9)	0 (0)
Alcohol use disorder	1 (1.6)	1 (2.2)	0 (0)
Anxiety disorder	1 (1.6)	1 (2.2)	0 (0)
Depression	1 (1.6)	1 (2.2)	0 (0)
Psychotic disorder	1 (1.6)	1 (2.2)	0 (0)
Reproductive system and breast disorders			
Benign prostatic hyperplasia	2 (3.3)	2 (4.4)	0 (0)
Respiratory, thoracic and mediastinal disorders			
All	5 (8.2)	4 (8.9)	1 (6.3)
Asthma	4 (6.6)	3 (6.7)	1 (6.3)
Chronic obstructive pulmonary disease	1 (1.6)	1 (2.2)	0 (0)
Vascular disorders			
Hypertension	28 (45.9)	20 (44.4)	8 (50.0)

**Abbreviations:** BMI, body mass index; NE, number of events; PT, preferred term; Q1–Q3, interquartile range; SD, standard deviation; SOC, system organ class. **Note:** Data are presented as *n* (%), mean (SD), or median (Q1–Q3). Percentages are based on the number of participants with available data. NE represents the number of medical history events (participants may have >1 event).

**Table 2:** Incidence of unsolicited adverse events during the first 28 days after an additional HXP-GPOVac dose and incidence of SAEs, medically attended adverse events (MAAEs), and AESIs throughout follow-up, by primary-series group and age stratum (safety population).

Adverse Events	Overall		18–59 yrs		≥60 yrs	
	HXP-GPOVac- Primed	BNT162b2- Primed	HXP-GPOVac- Primed	BNT162b2- Primed	HXP-GPOVac- Primed	BNT162b2- Primed
	(*N* = 167)	(*N* = 52)	(*N* = 135)	(*N* = 42)	(*N* = 32)	(*N* = 10)
Unsolicited adverse events (AE)—*n* (%)						
With one or more unsolicited AEs	7 (4.2)	2 (3.8)	5 (3.7)	0 (0)	2 (6.3)	2 (20.0)
(95% CI)	(1.7–8.4)	(0.5–13.2)	(1.2–8.4)	(0–8.4)	(0.8–20.8)	(2.5–55.6)
Mild (Grade 1)	6 (3.6)	1 (1.9)	4 (2.3)	0 (0)	2 (6.3)	1 (10.0)
Moderate (Grade 2)	0 (0)	1 (1.9)	0 (0)	0 (0)	0 (0)	1 (10.0)
Severe (Grade 3)	1 (0.6)	0 (0)	1 (0.6)	0 (0)	0 (0)	0 (0)
Potentially life-threatening (Grade 4)	0 (0)	0 (0)	0 (0)	0 (0)	0 (0)	0 (0)
Fatal (Grade 5)	0 (0)	0 (0)	0 (0)	0 (0)	0 (0)	0 (0)
None	160 (95.8)	50 (96.2)	130 (96.3)	42 (100)	30 (93.8)	8 (80.0)
(95% CI)	(91.6–98.3)	(86.8–99.5)	(91.6–98.8)	(91.6–100)	(79.2–99.2)	(44.4–97.5)
With vaccine-related unsolicited AEs	0 (0)	0 (0)	0 (0)	0 (0)	0 (0)	0 (0)
(95% CI)	(0–2.2)	(0–6.8)	(0–2.7)	(0–8.4)	(0–10.9)	(0–30.8)
Serious adverse events (SAE)—*n* (%)						
With one or more SAEs	1 (0.6)	0 (0)	1 (0.7)	0 (0)	0 (0)	0 (0)
(95% CI)	(0–3.3)	(0–6.8)	(0–4.1)	(0–8.4)	(0–10.9)	(0–30.8)
With no SAE	166 (99.4)	52 (100)	134 (99.3)	42 (100)	32 (100)	10 (100)
(95% CI)	(96.7–100)	(93.2–100)	(95.9–100)	(91.6–100)	(89.1–100)	(69.2–100)
With vaccine-related SAEs	0 (0)	0 (0)	0 (0)	0 (0)	0 (0)	0 (0)
(95% CI)	(0–2.2)	(0–6.8)	(0–2.7)	(0–8.4)	(0–10.9)	(0–30.8)
SAEs leading to death	0 (0)	0 (0)	0 (0)	0 (0)	0 (0)	0 (0)
(95% CI)	(0–2.2)	(0–6.8)	(0–2.7)	(0–8.4)	(0–10.9)	(0–30.8)
Medically attended adverse events (MAAE)—*n* (%)						
With one or more MAAEs	1 (0.6)	1 (1.9)	1 (0.7)	0 (0)	0 (0)	1 (10.0)
(95% CI)	(0–3.3)	(0–10.3)	(0–4.1)	(0–8.4)	(0–10.9)	(0.3–44.5)
With no MAAE	166 (99.4)	51 (98.1)	134 (99.3)	42 (100)	32 (100)	9 (90.0)
(95% CI)	(96.7–100)	(89.7–100)	(95.9–100)	(91.6–100)	(89.1–100)	(55.5–99.7)
With vaccine-related MAAEs	0 (0)	0 (0)	0 (0)	0 (0)	0 (0)	0 (0)
(95% CI)	(0–2.2)	(0–6.8)	(0–2.7)	(0–8.4)	(0–10.9)	(0–30.8)
Adverse events of special interest (AESI)—*n* (%)						
With one or more AESIs	0 (0)	0 (0)	0 (0)	0 (0)	0 (0)	0 (0)
(95% CI)	(0–2.2)	(0–6.8)	(0–2.7)	(0–8.4)	(0–10.9)	(0–30.8)
With no AESI	167 (100)	52 (100)	135 (100)	42 (100)	32 (100)	10 (100)
(95% CI)	(97.8–100)	(93.2–100)	(97.3–100)	(91.6–100)	(89.1–100)	(69.2–100)
With vaccine-related AESIs	0 (0)	0 (0)	0 (0)	0 (0)	0 (0)	0 (0)
(95% CI)	(0–2.2)	(0–6.8)	(0–2.7)	(0–8.4)	(0–10.9)	(0–30.8)
Serious adverse events (SAE) throughout the study—*n* (%)						
With one or more SAEs	8 (4.8)	1 (1.9)	8 (5.9)	1 (2.4)	0 (0)	0 (0)
(95% CI)	(2.1–9.2)	(0–10.3)	(2.6–11.3)	(0.1–12.6)	(0–10.9)	(0–30.8)
Mild (Grade 1)	0 (0)	0 (0)	0 (0)	0 (0)	0 (0)	0 (0)
Moderate (Grade 2)	0 (0)	0 (0)	0 (0)	0 (0)	0 (0)	0 (0)
Severe (Grade 3)	6 (3.6)	0 (0)	6 (4.4)	0 (0)	0 (0)	0 (0)
Potentially life-threatening (Grade 4)	0 (0)	0 (0)	0 (0)	0 (0)	0 (0)	0 (0)
Fatal (Grade 5)	2 (1.2)	1 (1.9)	2 (1.5)	1 (2.4)	0 (0)	0 (0)
With no SAE	159 (95.2)	51 (98.1)	127 (94.1)	41 (97.6)	32 (100)	10 (100)
(95% CI)	(90.8–97.9)	(89.7–100.0)	(88.7–97.4)	(87.4–99.9)	(89.1–100)	(69.2–100)
With vaccine-related SAEs	1 (0.6)	0 (0)	1 (0.7)	0 (0)	0 (0)	0 (0)
(95% CI)	(0.0–3.3)	(0–6.8)	(0.0–4.1)	(0–8.4)	(0–10.9)	(0–30.8)
Medically attended adverse events (MAAE) throughout the study—*n* (%)						
With one or more serious MAAEs	8 (4.8)	0 (0)	8 (5.9)	0 (0)	0 (0)	0 (0)
(95% CI)	(2.1–9.2)	(0–6.8)	(2.6–11.3)	(0–8.4)	(0–10.9)	(0–30.8)
Mild (Grade 1)	4 (2.4)	1 (1.9)	2 (1.5)	1 (2.4)	2 (6.3)	0 (0)
Moderate (Grade 2)	11 (6.6)	4 (7.7)	7 (5.2)	3 (7.1)	4 (12.5)	1 (10.0)
Severe (Grade 3)	6 (3.6)	0 (0)	6 (4.4)	0 (0)	0 (0)	0 (0)
Potentially life-threatening (Grade 4)	0 (0)	0 (0)	0 (0)	0 (0)	0 (0)	0 (0)
Fatal (Grade 5)	2 (1.2)	0 (0)	2 (1.5)	0 (0)	0 (0)	0 (0)
With no serious MAAE	159 (95.2)	52 (100)	127 (94.1)	42 (100)	32 (100)	10 (100)
(95% CI)	(90.8–97.9)	(93.2–100)	(88.7–97.4)	(91.6–100)	(89.1–100)	(69.2–100)
With vaccine-related MAAEs	0 (0)	0 (0)	0 (0)	0 (0)	0 (0)	0 (0)
(95% CI)	(0–2.2)	(0–6.8)	(0–2.7)	(0–8.4)	(0–10.9)	(0–30.8)
Adverse events of special interest (AESI) throughout the study—*n* (%)						
With one or more AESIs	0 (0)	0 (0)	0 (0)	0 (0)	0 (0)	0 (0)
(95% CI)	(0–2.2)	(0–6.8)	(0–2.7)	(0–8.4)	(0–10.9)	(0–30.8)
With no AESI	167 (100)	52 (100)	135 (100)	42 (100)	32 (100)	10 (100)
(95% CI)	(97.8–100)	(93.2–100)	(97.3–100)	(91.6–100)	(89.1–100)	(69.2–100)
With vaccine-related AESIs	0 (0)	0 (0)	0 (0)	0 (0)	0 (0)	0 (0)
(95% CI)	(0–2.2)	(0–6.8)	(0–2.7)	(0–8.4)	(0–10.9)	(0–30.8)

**Abbreviations:** AE, adverse event; AESI, adverse event of special interest; CI, confidence interval; MAAE, medically attended adverse event; MedDRA, Medical Dictionary for Regulatory Activities; SAE, serious adverse event; SOC, system organ class; PT, preferred term. **Note:** Data are *n* (%) of participants. Events are coded by MedDRA SOC and PT. Relatedness and seriousness were assessed by investigators. The one vaccine-related SAE summarized here is a fatal event (sudden cardiac death) assessed by the safety medical team as possibly related to vaccination; no SAE was assessed as probably or definitely related. Two-sided 95% CIs are exact (Clopper–Pearson).

**Table 3:** Geometric mean titer or concentration ratios of 50% neutralizing antibody titers measured by pseudovirus neutralization assay and anti-spike protein immunoglobulin G antibody titers measured by enzyme-linked immunosorbent assay at baseline, 14 days (D15), 3 months (D85), 6 months (D169) and 12 months (D337) after an additional HXP-GPOVac dose, stratified by age (per-protocol population).

	Overall	18–59 yrs	≥60 yrs
	HXP-GPOVac-Primed/BNT162b2-Primed	HXP-GPOVac-Primed/BNT162b2-Primed	HXP-GPOVac-Primed/BNT162b2-Primed
Baseline (D1)			
NT_50_ GMT ratio (95% CI)	0.60 (0.35–1.03)	0.47 (0.26–0.83)	1.74 (0.39–7.65)
Anti-S IgG GMC ratio (95% CI)	0.25 (0.15–0.41)	0.20 (0.12–0.34)	0.64 (0.17–2.47)
14 days after the vaccination (D15)			
NT_50_ GMT ratio (95% CI)	1.86 (1.35–2.58)	1.66 (1.19–2.32)	3.02 (1.22–7.46)
Anti-S IgG GMC ratio (95% CI)	0.96 (0.74–1.24)	0.90 (0.68–1.19)	1.22 (0.61–2.42)
3 months after the vaccination (D85)			
NT_50_ GMT ratio (95% CI)	0.92 (0.67–1.26)	0.87 (0.61–1.23)	1.14 (0.54–2.40)
Anti-S IgG GMC ratio (95% CI)	0.54 (0.39–0.73)	0.53 (0.38–0.75)	0.57 (0.27–1.21)
6 months after the vaccination (D169)			
NT_50_ GMT ratio (95% CI)	0.77 (0.54–1.08)	0.74 (0.52–1.07)	0.88 (0.34–2.27)
Anti-S IgG GMC ratio (95% CI)	0.42 (0.30–0.60)	0.42 (0.29–0.61)	0.41 (0.15–1.15)
12 months after the vaccination (D337)			
NT_50_ GMT ratio (95% CI)	0.64 (0.45–0.91)	0.62 (0.42–0.90)	0.71 (0.27–1.91)
Anti-S IgG GMC ratio (95% CI)	0.34 (0.24–0.49)	0.32 (0.21–0.47)	0.45 (0.18–1.11)

**Abbreviations:** PNA, pseudovirus neutralization assay; NT_50_, 50% neutralization titer; ELISA, enzyme-linked immunosorbent assay; S, spike; GMT, geometric mean titer; GMC, geometric mean concentration; BAU/mL, binding antibody units per milliliter; CI, confidence interval; D#, visit day (e.g., D1 = visit day 1, D29 = visit day 29, etc.). **Note:** Ratios are geometric means of individual response ratios. 95% CIs are calculated from log-transformed data.

## Data Availability

The data presented in this study are available on reasonable request from the corresponding author. The data are not publicly available due to participant privacy and sponsor restrictions.

## Supplementary Materials

The following supporting information can be downloaded at https://www.mdpi.com/article/10.3390/vaccines14080660/s1, Supplementary Table S1. Incidence of solicited local and systemic adverse events during the first 7 days after an additional HXP-GPOVac dose, stratified by primary-series group and age (safety population); Supplementary Table S2. Geometric mean titers or concentrations, geometric fold rises, and seroconversion rates of 50% neutralizing antibody titers measured by pseudovirus neutralization assay and anti-spike protein immunoglobulin G antibody titers measured by enzyme-linked immunosorbent assay at baseline, 14 days (D15), 3 months (D85), 6 months (D169) and 12 months (D337) after an additional HXP-GPOVac dose, stratified by primary-series group and age (per-protocol population); Supplementary Table S3. Cell-mediated immune responses to SARS-CoV-2 spike: IFN-γ and IL-5 ELISpot geometric mean concentrations and ratios in HXP-GPOVac-primed and BNT162b2-primed recipients at baseline (D1), 7 days (D8), 1 month (D29) and 6 months (D169) after an additional HXP-GPOVac dose, using SARS-CoV-2 peptides, stratified by primary-series group and age (per-protocol population); Supplementary Table S4. Incidence density of anti-N seropositivity (anti-N ≥ 1.1 among baseline anti-N–seronegative participants), symptomatic RT-PCR–confirmed COVID-19 during follow-up and SARS-CoV-2 sequencing results among RT-PCR–confirmed symptomatic COVID-19 cases during follow-up; Supplementary Table S5. Anti–SARS-CoV-2 nucleocapsid (anti-N) IgG over time after an additional HXP-GPOVac dose, by primary-series group and age stratum (per-protocol population); Supplementary Table S6. Severity grading scale for solicited adverse events.
