# Supplementary material for: Safety and Immunogenicity of an Additional Dose of Thailand Government Pharmaceutical Organization (GPO) Inactivated NDV-HXP-S COVID-19 Vaccine (HXP-GPOVac) Administered After Primary Vaccination with HXP-GPOVac or BNT162b2: An Open-Label Phase II Extension Trial in Thai Adults
Source: Vaccines (Basel). 2026 Jul 28;14(8):660. doi: 10.3390/vaccines14080660 (PMC13517118; doi:10.3390/vaccines14080660)
Supplement: Supplementary file 1 [file vaccines-14-00660-s001.zip › vaccines-4430040-supplementary.pdf]

Supplementary Materials

Supplementary information for: *Safety and immunogenicity of an additional dose of Thailand Government Pharmaceutical Organization (GPO) inactivated NDV-HXP-S COVID-19 vaccine (HXP-GPOVac) administered after primary vaccination with HXP-GPOVac or BNT162b2: an open-label phase II extension trial in Thai adults*

Supplementary tables

Supplementary Table S1 – Incidence of solicited local and systemic adverse events during the first 7 days after an additional HXP-GPOVac dose, stratified by primary-series group and age (safety population).

Adverse events	Overall		18 – 59 yrs		≥ 60 yrs	
	HXP-GPOVac-primed (*N* = 167)	BNT162b2-primed (*N* = 52)	HXP-GPOVac-primed (*N* = 135)	BNT162b2-primed (*N* = 42)	HXP-GPOVac-primed (*N* = 32)	BNT162b2-primed (*N* = 10)
Solicited local adverse events						
Any local reaction – *n* (%)	37 (22.2)	14 (26.9)	27 (20.0)	12 (28.6)	10 (31.3)	2 (20.0)
(95% CI)	(16.1–29.2)	(15.6–41.0)	(13.6–27.7)	(15.7–44.6)	(16.1–50.0)	(2.5–55.6)
Pain or Tenderness						
With one or more – *n* (%)	37 (22.2)	14 (26.9)	27 (20.0)	12 (28.6)	10 (31.3)	2 (20.0)
(95% CI)	(16.1–29.2)	(15.6–41.0)	(13.6–27.7)	(15.7–44.6)	(16.1–50.0)	(2.5–55.6)
None – *n* (%)	130 (77.8)	38 (73.1)	108 (80.0)	30 (71.4)	22 (68.8)	8 (80.0)
(95% CI)	(70.8–83.9)	(59.0–84.4)	(72.3–86.4)	(55.4–84.3)	(50.0–83.9)	(44.4–97.5)
Mild (Grade 1) – *n* (%)	22 (13.2)	9 (17.3)	16 (11.9)	7 (16.7)	6 (18.8)	2 (20.0)
(95% CI)	(8.4–19.3)	(8.2–30.3)	(6.9–18.5)	(7.0–31.4)	(7.2–36.4)	(2.5–55.6)
Moderate (Grade 2) – *n* (%)	15 (9.0)	5 (9.6)	11 (8.1)	5 (11.9)	4 (12.5)	0 (0)
(95% CI)	(5.1–14.4)	(3.2–21.0)	(4.1–14.1)	(4.0–25.6)	(3.5–29.0)	(0–30.8)
Severe (Grade 3) – *n* (%)	0 (0)	0 (0)	0 (0)	0 (0)	0 (0)	0 (0)
(95% CI)	(0–2.2)	(0–6.8)	(0–2.7)	(0–8.4)	(0–10.9)	(0–30.8)
Potentially life-threatening (Grade 4) – *n* (%)	0 (0)	0 (0)	0 (0)	0 (0)	0 (0)	0 (0)
(95% CI)	(0–2.2)	(0–6.8)	(0–2.7)	(0–8.4)	(0–10.9)	(0–30.8)

Adverse events	Overall		18 – 59 yrs		≥ 60 yrs	
	HXP-GPOVac-primed	BNT162b2-primed	HXP-GPOVac-primed	BNT162b2-primed	HXP-GPOVac-primed	BNT162b2-primed
	(*N* = 167)	(*N* = 52)	(*N* = 135)	(*N* = 42)	(*N* = 32)	(*N* = 10)
Swelling or Induration						
With one or more – *n* (%)	0 (0)	0 (0)	0 (0)	0 (0)	0 (0)	0 (0)
(95% CI)	(0–2.2)	(0–6.8)	(0–2.7)	(0–8.4)	(0–10.9)	(0–30.8)
None – *n* (%)	167 (100)	52 (100)	135 (100)	42 (100)	32 (100)	10 (100)
(95% CI)	(97.8–100)	(93.2–100)	(97.3–100)	(91.6–100)	(89.1–100)	(69.2–100)
Mild (Grade 1) – *n* (%)	0 (0)	0 (0)	0 (0)	0 (0)	0 (0)	0 (0)
(95% CI)	(0–2.2)	(0–6.8)	(0–2.7)	(0–8.4)	(0–10.9)	(0–30.8)
Moderate (Grade 2) – *n* (%)	0 (0)	0 (0)	0 (0)	0 (0)	0 (0)	0 (0)
(95% CI)	(0–2.2)	(0–6.8)	(0–2.7)	(0–8.4)	(0–10.9)	(0–30.8)
Severe (Grade 3) – *n* (%)	0 (0)	0 (0)	0 (0)	0 (0)	0 (0)	0 (0)
(95% CI)	(0–2.2)	(0–6.8)	(0–2.7)	(0–8.4)	(0–10.9)	(0–30.8)
Potentially life-threatening (Grade 4) – *n* (%)	0 (0)	0 (0)	0 (0)	0 (0)	0 (0)	0 (0)
(95% CI)	(0–2.2)	(0–6.8)	(0–2.7)	(0–8.4)	(0–10.9)	(0–30.8)
Erythema						
With one or more – *n* (%)	0 (0)	0 (0)	0 (0)	0 (0)	0 (0)	0 (0)
(95% CI)	(0–2.2)	(0–6.8)	(0–2.7)	(0–8.4)	(0–10.9)	(0–30.8)
None – *n* (%)	167 (100)	52 (100)	135 (100)	42 (100)	32 (100)	10 (100)
(95% CI)	(97.8–100)	(93.2–100)	(97.3–100)	(91.6–100)	(89.1–100)	(69.2–100)
Mild (Grade 1) – *n* (%)	0 (0)	0 (0)	0 (0)	0 (0)	0 (0)	0 (0)
(95% CI)	(0–2.2)	(0–6.8)	(0–2.7)	(0–8.4)	(0–10.9)	(0–30.8)
Moderate (Grade 2) – *n* (%)	0 (0)	0 (0)	0 (0)	0 (0)	0 (0)	0 (0)
(95% CI)	(0–2.2)	(0–6.8)	(0–2.7)	(0–8.4)	(0–10.9)	(0–30.8)
Severe (Grade 3) – *n* (%)	0 (0)	0 (0)	0 (0)	0 (0)	0 (0)	0 (0)
(95% CI)	(0–2.2)	(0–6.8)	(0–2.7)	(0–8.4)	(0–10.9)	(0–30.8)
Potentially life-threatening (Grade 4) – *n* (%)	0 (0)	0 (0)	0 (0)	0 (0)	0 (0)	0 (0)
(95% CI)	(0–2.2)	(0–6.8)	(0–2.7)	(0–8.4)	(0–10.9)	(0–30.8)

Adverse events	Overall		18 – 59 yrs		≥ 60 yrs	
	HXP-GPOVac-primed (*N* = 167)	BNT162b2-primed (*N* = 52)	HXP-GPOVac-primed (*N* = 135)	BNT162b2-primed (*N* = 42)	HXP-GPOVac-primed (*N* = 32)	BNT162b2-primed (*N* = 10)
Solicited systemic adverse events						
Any systemic reaction – *n* (%)	18 (10.8)	7 (13.5)	13 (9.6)	5 (11.9)	5 (15.6)	2 (20.0)
(95% CI)	(6.5–16.5)	(5.6–25.8)	(5.2–15.9)	(4.0–25.6)	(5.3–32.8)	(2.5–55.6)
Fever (≥38°C)						
With one or more – *n* (%)	3 (1.8)	1 (1.9)	1 (0.7)	1 (2.4)	2 (6.3)	0 (0)
(95% CI)	(0.4–5.2)	(0–10.3)	(0–4.1)	(0.1–12.6)	(0.8–20.8)	(0–30.8)
None – *n* (%)	164 (98.2)	51 (98.1)	134 (99.3)	41 (97.6)	30 (93.8)	10 (100)
(95% CI)	(94.8–99.6)	(89.7–100)	(95.9–100)	(87.4–99.9)	(79.2–99.2)	(69.2–100)
Mild (Grade 1) – *n* (%)	3 (1.8)	0 (0)	1 (0.7)	0 (0)	2 (6.3)	0 (0)
(95% CI)	(0.4–5.2)	(0–6.8)	(0–4.1)	(0–8.4)	(0.8–20.8)	(0–30.8)
Moderate (Grade 2) – *n* (%)	0 (0)	1 (1.9)	0 (0)	1 (2.4)	0 (0)	0 (0)
(95% CI)	(0–2.2)	(0–10.3)	(0–2.7)	(0.1–12.6)	(0–10.9)	(0–30.8)
Severe (Grade 3) – *n* (%)	0 (0)	0 (0)	0 (0)	0 (0)	0 (0)	0 (0)
(95% CI)	(0–2.2)	(0–6.8)	(0–2.7)	(0–8.4)	(0–10.9)	(0–30.8)
Potentially life-threatening (Grade 4) – *n* (%)	0 (0)	0 (0)	0 (0)	0 (0)	0 (0)	0 (0)
(95% CI)	(0–2.2)	(0–6.8)	(0–2.7)	(0–8.4)	(0–10.9)	(0–30.8)
Headache						
With one or more – *n* (%)	11 (6.6)	1 (1.9)	7 (5.2)	1 (2.4)	4 (12.5)	0 (0)
(95% CI)	(3.3–11.5)	(0–10.3)	(2.1–10.4)	(0.1–12.6)	(3.5–29.0)	(0–30.8)
None – *n* (%)	156 (93.4)	51 (98.1)	128 (94.8)	41 (97.6)	28 (87.5)	10 (100)
(95% CI)	(88.5–96.7)	(89.7–100)	(89.6–97.9)	(87.4–99.9)	(71.0–96.5)	(69.2–100)
Mild (Grade 1) – *n* (%)	10 (6.0)	0 (0)	6 (4.4)	0 (0)	4 (12.5)	0 (0)
(95% CI)	(2.9–10.7)	(0–6.8)	(1.6–9.4)	(0–8.4)	(3.5–29.0)	(0–30.8)
Moderate (Grade 2) – *n* (%)	1 (0.6)	1 (1.9)	1 (0.7)	1 (2.4)	0 (0)	0 (0)
(95% CI)	(0–3.3)	(0–10.3)	(0–4.1)	(0.1–12.6)	(0–10.9)	(0–30.8)
Severe (Grade 3) – *n* (%)	0 (0)	0 (0)	0 (0)	0 (0)	0 (0)	0 (0)

Adverse events	Overall		18 – 59 yrs		≥ 60 yrs	
	HXP-GPOVac-primed (*N* = 167)	BNT162b2-primed (*N* = 52)	HXP-GPOVac-primed (*N* = 135)	BNT162b2-primed (*N* = 42)	HXP-GPOVac-primed (*N* = 32)	BNT162b2-primed (*N* = 10)
(95% CI)	(0–2.2)	(0–6.8)	(0–2.7)	(0–8.4)	(0–10.9)	(0–30.8)
Potentially life-threatening (Grade 4) – *n* (%)	0 (0)	0 (0)	0 (0)	0 (0)	0 (0)	0 (0)
(95% CI)	(0–2.2)	(0–6.8)	(0–2.7)	(0–8.4)	(0–10.9)	(0–30.8)
Fatigue or Malaise						
With one or more – *n* (%)	13 (7.8)	4 (7.7)	8 (5.9)	2 (4.8)	5 (15.6)	2 (20.0)
(95% CI)	(4.2–12.9)	(2.1–18.5)	(2.6–11.3)	(0.6–16.2)	(5.3–32.8)	(2.5–55.6)
None – *n* (%)	154 (92.2)	48 (92.3)	127 (94.1)	40 (95.2)	27 (84.4)	8 (80.0)
(95% CI)	(87.1–95.8)	(81.5–97.9)	(88.7–97.4)	(83.8–99.4)	(67.2–94.7)	(44.4–97.5)
Mild (Grade 1) – *n* (%)	11 (6.6)	2 (3.8)	7 (5.2)	1 (2.4)	4 (12.5)	1 (10.0)
(95% CI)	(3.3–11.5)	(0.5–13.2)	(2.1–10.4)	(0.1–12.6)	(3.5–29.0)	(0.3–44.5)
Moderate (Grade 2) – *n* (%)	2 (1.2)	2 (3.8)	1 (0.7)	1 (2.4)	1 (3.1)	1 (10.0)
(95% CI)	(0.1–4.3)	(0.5–13.2)	(0–4.1)	(0.1–12.6)	(0.1–16.2)	(0.3–44.5)
Severe (Grade 3) – *n* (%)	0 (0)	0 (0)	0 (0)	0 (0)	0 (0)	0 (0)
(95% CI)	(0–2.2)	(0–6.8)	(0–2.7)	(0–8.4)	(0–10.9)	(0–30.8)
Potentially life-threatening (Grade 4) – *n* (%)	0 (0)	0 (0)	0 (0)	0 (0)	0 (0)	0 (0)
(95% CI)	(0–2.2)	(0–6.8)	(0–2.7)	(0–8.4)	(0–10.9)	(0–30.8)
Myalgia						
With one or more – *n* (%)	16 (9.6)	5 (9.6)	12 (8.9)	4 (9.5)	4 (12.5)	1 (10.0)
(95% CI)	(5.6–15.1)	(3.2–21.0)	(4.7–15.0)	(2.7–22.6)	(3.5–29.0)	(0.3–44.5)
None – *n* (%)	151 (90.4)	47 (90.4)	123 (91.1)	38 (90.5)	28 (87.5)	9 (90.0)
(95% CI)	(84.9–94.4)	(79.0–96.8)	(85.0–95.3)	(77.4–97.3)	(71.0–96.5)	(55.5–99.7)
Mild (Grade 1) – *n* (%)	13 (7.8)	5 (9.6)	10 (7.4)	4 (9.5)	3 (9.4)	1 (10.0)
(95% CI)	(4.2–12.9)	(3.2–21.0)	(3.6–13.2)	(2.7–22.6)	(2.0–25.0)	(0.3–44.5)
Moderate (Grade 2) – *n* (%)	3 (1.8)	0 (0)	2 (1.5)	0 (0)	1 (3.1)	0 (0)
(95% CI)	(0.4–5.2)	(0–6.8)	(0.2–5.2)	(0–8.4)	(0.1–16.2)	(0–30.8)
Severe (Grade 3) – *n* (%)	0 (0)	0 (0)	0 (0)	0 (0)	0 (0)	0 (0)

Adverse events	Overall		18 – 59 yrs		≥ 60 yrs	
	HXP-GPOVac-primed (*N* = 167)	BNT162b2-primed (*N* = 52)	HXP-GPOVac-primed (*N* = 135)	BNT162b2-primed (*N* = 42)	HXP-GPOVac-primed (*N* = 32)	BNT162b2-primed (*N* = 10)
(95% CI)	(0–2.2)	(0–6.8)	(0–2.7)	(0–8.4)	(0–10.9)	(0–30.8)
Potentially life-threatening (Grade 4) – *n* (%)	0 (0)	0 (0)	0 (0)	0 (0)	0 (0)	0 (0)
(95% CI)	(0–2.2)	(0–6.8)	(0–2.7)	(0–8.4)	(0–10.9)	(0–30.8)
Arthralgia						
With one or more – *n* (%)	7 (4.2)	1 (1.9)	5 (3.7)	0 (0)	2 (6.3)	1 (10.0)
(95% CI)	(1.7–8.4)	(0–10.3)	(1.2–8.4)	(0–8.4)	(0.8–20.8)	(0.3–44.5)
None – *n* (%)	160 (95.8)	51 (98.1)	130 (96.3)	42 (100)	30 (93.8)	9 (90.0)
(95% CI)	(91.6–98.3)	(89.7–100)	(91.6–98.8)	(91.6–100)	(79.2–99.2)	(55.5–99.7)
Mild (Grade 1) – *n* (%)	6 (3.6)	1 (1.9)	5 (3.7)	0 (0)	1 (3.1)	1 (10.0)
(95% CI)	(1.3–7.7)	(0–10.3)	(1.2–8.4)	(0–8.4)	(0.1–16.2)	(0.3–44.5)
Moderate (Grade 2) – *n* (%)	1 (0.6)	0 (0)	0 (0)	0 (0)	1 (3.1)	0 (0)
(95% CI)	(0–3.3)	(0–6.8)	(0–2.7)	(0–8.4)	(0.1–16.2)	(0–30.8)
Severe (Grade 3) – *n* (%)	0 (0)	0 (0)	0 (0)	0 (0)	0 (0)	0 (0)
(95% CI)	(0–2.2)	(0–6.8)	(0–2.7)	(0–8.4)	(0–10.9)	(0–30.8)
Potentially life-threatening (Grade 4) – *n* (%)	0 (0)	0 (0)	0 (0)	0 (0)	0 (0)	0 (0)
(95% CI)	(0–2.2)	(0–6.8)	(0–2.7)	(0–8.4)	(0–10.9)	(0–30.8)
Nausea or Vomiting						
With one or more – *n* (%)	1 (0.6)	0 (0)	1 (0.7)	0 (0)	0 (0)	0 (0)
(95% CI)	(0–3.3)	(0–6.8)	(0–4.1)	(0–8.4)	(0–10.9)	(0–30.8)
None – *n* (%)	166 (99.4)	52 (100)	134 (99.3)	42 (100)	32 (100)	10 (100)
(95% CI)	(96.7–100.0)	(93.2–100)	(95.9–100)	(91.6–100)	(89.1–100)	(69.2–100)
Mild (Grade 1) – *n* (%)	1 (0.6)	0 (0)	1 (0.7)	0 (0)	0 (0)	0 (0)
(95% CI)	(0–3.3)	(0–6.8)	(0–4.1)	(0–8.4)	(0–10.9)	(0–30.8)
Moderate (Grade 2) – *n* (%)	0 (0)	0 (0)	0 (0)	0 (0)	0 (0)	0 (0)
(95% CI)	(0–2.2)	(0–6.8)	(0–2.7)	(0–8.4)	(0–10.9)	(0–30.8)
Severe (Grade 3) – *n* (%)	0 (0)	0 (0)	0 (0)	0 (0)	0 (0)	0 (0)

Adverse events	Overall		18 – 59 yrs		≥ 60 yrs	
	HXP-GPOVac-primed (*N* = 167)	BNT162b2-primed (*N* = 52)	HXP-GPOVac-primed (*N* = 135)	BNT162b2-primed (*N* = 42)	HXP-GPOVac-primed (*N* = 32)	BNT162b2-primed (*N* = 10)
(95% CI)	(0–2.2)	(0–6.8)	(0–2.7)	(0–8.4)	(0–10.9)	(0–30.8)
Potentially life-threatening (Grade 4) – *n* (%)	0 (0)	0 (0)	0 (0)	0 (0)	0 (0)	0 (0)
(95% CI)	(0–2.2)	(0–6.8)	(0–2.7)	(0–8.4)	(0–10.9)	(0–30.8)

Abbreviations: AE, adverse event; CI, confidence interval.

Note: Data are *n* (%) of participants by maximum grade (Grade 1 = mild, 2 = moderate, 3 = severe, 4 = life-threatening). Percentages are by dose. 95% CIs by Clopper–Pearson.

Supplementary Table S2 – Geometric mean titers or concentrations, geometric fold rises, and seroconversion rates of 50% neutralizing antibody titers measured by pseudovirus neutralization assay and anti-spike protein immunoglobulin G antibody titers measured by enzyme-linked immunosorbent assay at baseline, 14 days (D15), 3 months (D85), 6 months (D169) and 12 months (D337) after an additional HXP-GPOVac dose, stratified by primary-series group and age (per-protocol population).

	Overall		18 – 59 yrs		≥ 60 yrs	
	HXP-GPOVac-primed (*N* = 167)	BNT162b2-primed (*N* = 52)	HXP-GPOVac-primed (*N* = 135)	BNT162b2-primed (*N* = 42)	HXP-GPOVac-primed (*N* = 32)	BNT162b2-primed (*N* = 10)
Baseline (D1) – *n*	167	52	135	42	32	10
PNA NT_{50} GMT – Reciprocal dilution	46.33	77.25	43.29	92.95	61.68	35.52
(95% CI)	(34.79–61.69)	(55.80–106.94)	(32.03–58.51)	(64.11–134.75)	(27.19–139.96)	(22.19–56.86)
ELISA Anti-S IgG GMC – BAU/mL	48.79	194.48	45.4	226.42	66.1	102.67
(95% CI)	(37.54–63.42)	(148.26–255.10)	(34.44–59.86)	(166.11–308.64)	(31.41–139.11)	(68.47–153.96)
14 days post vaccination (D15) – *n*	165	52	133	42	32	10
PNA NT_{50} GMT – Reciprocal dilution	1569.04	841.34	1406.93	847.11	2468.81	817.54
(95% CI)	(1329.51–1851.73)	(659.86–1072.74)	(1189.20–1664.51)	(639.47–1122.17)	(1524.06–3999.20)	(468.48–1426.68)
PNA NT_{50} GMFR – fold	28.24	10.89	26.8	9.11	35.15	23.02
(95% CI)	(20.87–38.22)	(7.82–15.17)	(19.46–36.89)	(6.30–13.19)	(14.93–82.75)	(11.85–44.70)
PNA NT_{50} SCR – *n* (%)	130 (78.8)	40 (76.9)	106 (79.7)	30 (71.4)	24 (75.0)	10 (100.0)

	Overall		18 – 59 yrs		≥ 60 yrs	
	HXP-GPOVac-primed (*N* = 167)	BNT162b2-primed (*N* = 52)	HXP-GPOVac-primed (*N* = 135)	BNT162b2-primed (*N* = 42)	HXP-GPOVac-primed (*N* = 32)	BNT162b2-primed (*N* = 10)
(95% CI)	(71.8–84.8)	(63.2–87.5)	(71.9–86.2)	(55.4–84.3)	(56.6–88.5)	(69.2–100.0)
ELISA Anti-S IgG GMC – BAU/mL	1480.14	1547.88	1380.37	1529.59	1978.15	1627.09
(95% CI)	(1296.90–1689.27)	(1270.98–1885.10)	(1201.93–1585.30)	(1217.07–1922.37)	(1372.04–2852.02)	(1043.19–2537.81)
ELISA Anti-S IgG GMFR – fold	28.11	7.96	27.98	6.76	28.66	15.85
(95% CI)	(21.66–36.49)	(6.01–10.54)	(21.20–36.93)	(4.92–9.27)	(13.83–59.39)	(10.07–24.95)
ELISA Anti-S IgG SCR – *n* (%)	138 (83.6)	37 (71.2)	112 (84.2)	27 (64.3)	26 (81.3)	10 (100.0)
(95% CI)	(77.1–88.9)	(56.9–82.9)	(76.9–90.0)	(48.0–78.4)	(63.6-92.8)	(69.2–100.0)
3 months post vaccination (D85) – *n*	161	51	129	41	32	10
PNA NT_{50} GMT – Reciprocal dilution	465.56	506.93	443.74	509.92	564.93	494.85
(95% CI)	(400.65–540.99)	(376.72–682.15)	(375.95–523.76)	(363.50–715.33)	(392.76–812.56)	(235.46–1039.96)
PNA NT_{50} GMFR – fold	8.42	6.59	8.52	5.49	8.04	13.93
(95% CI)	(6.34–11.19)	(4.26–10.22)	(6.27–11.58)	(3.38–8.92)	(3.80–17.03)	(4.81–40.37)
PNA NT_{50} SCR – *n* (%)	104 (64.6)	27 (52.9)	83 (64.3)	20 (48.8)	21 (65.6)	7 (70.0)
(95% CI)	(56.7–72.0)	(38.5–67.1)	(55.4–72.6)	(32.9–64.9)	(46.8–81.4)	(34.8–93.3)
ELISA Anti-S IgG GMC – BAU/mL	604.12	1124.75	588.04	1110.08	673.56	1186.95
(95% CI)	(518.40–704.02)	(854.33–1480.76)	(497.60–694.92)	(806.08–1528.73)	(455.34–996.35)	(641.80–2195.13)
ELISA Anti-S IgG GMFR – fold	11.54	5.74	12.03	4.84	9.76	11.56
(95% CI)	(8.87–15.00)	(3.96–8.32)	(9.07–15.95)	(3.18–7.36)	(4.84–19.68)	(5.44–24.59)
ELISA Anti-S IgG SCR – *n* (%)	116 (72.0)	27 (52.9)	95 (73.6)	19 (46.3)	21 (65.6)	8 (80.0)
(95% CI)	(64.4–78.8)	(38.5–67.1)	(65.2–81.0)	(30.7–62.6)	(46.8–81.4)	(44.4–97.5)
6 months post vaccination (D169) – *n*	158	48	127	39	31	9
PNA NT_{50} GMT – Reciprocal dilution	230.15	300.18	229.3	308.61	233.69	266.25
(95% CI)	(195.20–271.37)	(220.77–408.16)	(192.87–272.60)	(217.34–438.19)	(145.36–375.69)	(123.32–574.86)
PNA NT_{50} GMFR – fold	4.11	3.88	4.38	3.34	3.17	7.46
(95% CI)	(3.13–5.40)	(2.46–6.13)	(3.30–5.82)	(2.01–5.55)	(1.43–7.03)	(2.35–23.72)
PNA NT_{50} SCR – *n* (%)	82 (51.9)	23 (47.9)	68 (53.5)	17 (43.6)	14 (45.2)	6 (66.7)

	Overall		18 – 59 yrs		≥ 60 yrs	
	HXP-GPOVac-primed (*N* = 167)	BNT162b2-primed (*N* = 52)	HXP-GPOVac-primed (*N* = 135)	BNT162b2-primed (*N* = 42)	HXP-GPOVac-primed (*N* = 32)	BNT162b2-primed (*N* = 10)
(95% CI)	(43.8–59.9)	(33.3–62.8)	(44.5–62.4)	(27.8–60.4)	(27.3–64.0)	(29.9–92.5)
ELISA Anti-S IgG GMC – BAU/mL	286.81	682.18	295.56	699.28	253.58	612.77
(95% CI)	(242.05–339.85)	(492.40–945.09)	(248.50–351.53)	(479.46–1019.88)	(151.22–425.23)	(289.57–1296.67)
ELISA Anti-S IgG GMFR – fold	5.46	3.43	6.05	3.03	3.61	5.91
(95% CI)	(4.19–7.13)	(2.24–5.25)	(4.62–7.92)	(1.86–4.93)	(1.61–8.12)	(2.35–14.87)
ELISA Anti-S IgG SCR – *n* (%)	87 (55.1)	17 (35.4)	74 (58.3)	11 (28.2)	13 (41.9)	6 (66.7)
(95% CI)	(47.0–63.0)	(22.2–50.5)	(49.2–67.0)	(15.0–44.9)	(24.5–60.9)	(29.9–92.5)
12 months post vaccination (D337) – *n*	158	49	126	39	32	10
PNA NT_{50} GMT – Reciprocal dilution	130.28	205.06	128.99	209.01	135.45	190.35
(95% CI)	(110.10–154.15)	(145.95–288.10)	(108.68–153.11)	(140.84–310.17)	(81.38–225.46)	(86.80–417.45)
PNA NT_{50} GMFR – fold	2.44	2.87	2.6	2.45	1.93	5.36
(95% CI)	(1.87–3.20)	(1.76–4.69)	(1.97–3.43)	(1.40–4.28)	(0.87–4.27)	(1.77–16.25)
PNA NT_{50} SCR – *n* (%)	63 (39.9)	16 (32.7)	52 (41.3)	10 (25.6)	11 (34.4)	6 (60.0)
(95% CI)	(32.2–48.0)	(19.9–47.5)	(32.6–50.4)	(13.0–42.1)	(18.6–53.2)	(26.2–87.8)
ELISA Anti-S IgG GMC – BAU/mL	177.8	521.63	174.82	549.65	190.03	425.34
(95% CI)	(149.86–210.93)	(367.75–739.91)	(145.82–209.58)	(363.84–830.34)	(118.47–304.81)	(207.83–870.50)
ELISA Anti-S IgG GMFR – fold	3.56	2.79	3.8	2.52	2.75	4.14
(95% CI)	(2.74–4.63)	(1.80–4.32)	(2.91–4.96)	(1.51–4.20)	(1.25–6.05)	(1.66–10.34)
ELISA SCR analysis population – *n* (%)	137	46	113	36	24	10
ELISA Anti-S IgG SCR – *n* (%)	71 (51.8)	18 (39.1)	60 (53.1)	12 (33.3)	11 (45.8)	6 (60.0)
(95% CI)	(43.1–60.4)	(25.1–54.6)	(43.5–62.5)	(18.6–51.0)	(25.6–67.2)	(26.2–87.8)

Abbreviations: PNA, pseudovirus neutralization assay; NT_{50}, 50% neutralization titer; ELISA, enzyme-linked immunosorbent assay; S, spike; GMT, geometric mean titer; GMC, geometric mean concentration; GMFR, geometric mean fold rise; SCR, seroconversion rate; BAU/mL, binding antibody units per milliliter; CI, confidence interval; LLOQ, lower limit of quantification; PP, per-protocol; D#, study day (e.g., D1 = Day 1, D29 = Day 29, etc.).

Note: GMTs, GMCs, and GMFRs are calculated from log-transformed values and back-transformed with two-sided 95% CIs. Titers < LLOQ (10) and concentrations < LLOQ (6.3 BAU/mL) were imputed as LLOQ/2. SCR is defined as ≥4-fold rise from baseline; 95% CIs by Clopper–Pearson.

Supplementary Table S3 – Cell-mediated immune responses to SARS-CoV-2 spike: IFN-γ and IL-5 ELISpot geometric mean concentrations and ratios in HXP-GPOVac-primed and BNT162b2-primed recipients at baseline (D1), 7 days (D8), 1 month (D29) and 6 months (D169) after an additional HXP-GPOVac dose, using SARS-CoV-2 peptides, stratified by primary-series group and age (per-protocol population).

	Vaccine group	
	HXP-GPOVac-primed	BNT162b2-primed
Baseline (D1) – *n*	27	9
SARS-CoV-2 DMSO		
IFN-gamma ELISpot		
GMC (95% CI) – SFU/10^6 PBMCs	5.41 (3.73–7.84)	4.08 (2.45–6.81)
Median (Min, Max)	3.32 (0, 35.00)	2.72 (0, 13.33)
IL-5 ELISpot		
GMC (95% CI) – SFU/10^6 PBMCs	4.80 (3.48–6.64)	6.05 (3.20–11.44)
Median (Min, Max)	5.00 (0, 21.67)	6.69 (0, 30.00)
IFN-gamma ELISpot / IL-5 ELISpot ratio		
GMC (95% CI)	0.90 (0.46–1.75)	0.48 (0.15–1.51)
Median (Min, Max)	1.17 (0.06, 12.98)	1.00 (0.03, 4.00)
SARS-CoV-2 Peptide 1		
IFN-gamma ELISpot		
GMC (95% CI) – SFU/10^6 PBMCs	31.03 (17.50–55.01)	11.84 (2.68–52.24)
Median (Min, Max)	46.53 (0, 173.33)	2.72 (0, 126.670)
IL-5 ELISpot		
GMC (95% CI) – SFU/10^6 PBMCs	9.18 (6.30–13.39)	13.04 (7.51–22.64)
Median (Min, Max)	11.70 (0, 43.33)	13.33 (0, 35.00)
IFN-gamma ELISpot / IL-5 ELISpot ratio		
GMC (95% CI)	3.03 (1.28–7.16)	0.65 (0.11–3.75)
Median (Min, Max)	5.54 (0.02, 52.05)	1.00 (0.05, 10.71)
SARS-CoV-2 Peptide 2		

	Vaccine group	
	HXP-GPOVac-primed	BNT162b2-primed
IFN-gamma ELISpot		
GMC (95% CI) – SFU/10^6 PBMCs	36.82 (18.94–71.57)	10.09 (2.99–34.04)
Median (Min, Max)	64.72 (0, 276.67)	5.00 (0, 96.67)
IL-5 ELISpot		
GMC (95% CI) – SFU/10^6 PBMCs	11.15 (8.06–15.41)	12.25 (7.40–20.29)
Median (Min, Max)	13.33 (1.67, 45.00)	11.70 (0, 25.00)
IFN-gamma ELISpot / IL-5 ELISpot ratio		
GMC (95% CI)	2.85 (1.27–6.38)	0.66 (0.16–2.73)
Median (Min, Max)	5.75 (0.06, 34.93)	1.00 (0.04, 5.80)
SARS-CoV-2 PHA		
IFN-gamma ELISpot		
GMC (95% CI) – SFU/10^6 PBMCs	591.68 (265.47–1318.73)	118.71 (11.07–1272.88)
Median (Min, Max)	1312.91 (0, 2166.67)	1248.88 (0, 1728.33)
IL-5 ELISpot		
GMC (95% CI) – SFU/10^6 PBMCs	187.14 (93.84–373.20)	117.27 (26.88–511.56)
Median (Min, Max)	307.97 (3.33, 851.67)	196.37 (10, 870.00)
IFN-gamma ELISpot / IL-5 ELISpot ratio		
GMC (95% CI)	2.83 (1.48–5.44)	0.73 (0.17–3.18)
Median (Min, Max)	2.59 (0.05, 202.20)	2.00 (0.04, 6.38)
7 days after the vaccination (D8) – *n*	27	9
SARS-CoV-2 DMSO		
IFN-gamma ELISpot		
GMC (95% CI) – SFU/10^6 PBMCs	5.10 (3.56–7.29)	4.25 (1.95–9.24)
Median (Min, Max)	3.32 (0, 81.67)	2.72 (0, 36.67)
IL-5 ELISpot		
GMC (95% CI) – SFU/10^6 PBMCs	4.51 (3.34–6.09)	5.06 (2.22–11.55)
Median (Min, Max)	3.32 (0, 21.67)	3.32 (0, 20.00)

	Vaccine group	
	HXP-GPOVac-primed	BNT162b2-primed
IFN-gamma ELISpot / IL-5 ELISpot ratio		
GMC (95% CI)	0.97 (0.50–1.90)	0.54 (0.13–2.31)
Median (Min, Max)	0.83 (0.08, 81.67)	0.60 (0.05, 36.67)
SARS-CoV-2 Peptide 1		
IFN-gamma ELISpot		
GMC (95% CI) – SFU/10^6 PBMCs	34.01 (19.11–60.53)	58.69 (13.75–250.49)
Median (Min, Max)	51.42 (0, 543.33)	104.58 (3.33, 406.67)
IL-5 ELISpot		
GMC (95% CI) – SFU/10^6 PBMCs	8.06 (5.66–11.48)	13.69 (9.37–19.99)
Median (Min, Max)	8.33 (0, 38.33)	13.33 (6.67, 36.67)
IFN-gamma ELISpot / IL-5 ELISpot ratio		
GMC (95% CI)	4.07 (2.15–7.69)	4.30 (1.02–18.01)
Median (Min, Max)	3.88 (0.12, 81.46)	4.75 (0.25, 34.01)
SARS-CoV-2 Peptide 2		
IFN-gamma ELISpot		
GMC (95% CI) – SFU/10^6 PBMCs	42.58 (22.84–79.41)	66.02 (19.25–226.46)
Median (Min, Max)	70.11 (0, 348.33)	75.19 (0, 288.33)
IL-5 ELISpot		
GMC (95% CI) – SFU/10^6 PBMCs	9.06 (6.36–12.90)	14.22 (9.38–21.56)
Median (Min, Max)	9.97 (0, 75.00)	13.33 (6.67, 31.67)
IFN-gamma ELISpot / IL-5 ELISpot ratio		
GMC (95% CI)	4.88 (2.60–9.15)	4.16 (1.05–16.53)
Median (Min, Max)	6.01 (0.08, 73.33)	5.43 (0.15, 43.23)
SARS-CoV-2 PHA		
IFN-gamma ELISpot		
GMC (95% CI) – SFU/10^6 PBMCs	621.78 (247.73–1560.61)	673.32 (117.09–3871.84)
Median (Min, Max)	1571.84 (0, 2203.33)	1408.10 (1.67–2505.00)

	Vaccine group	
	HXP-GPOVac-primed	BNT162b2-primed
IL-5 ELISpot		
GMC (95% CI) – SFU/10^6 PBMCs	143.60 (64.53–319.58)	336.60 (119.41–948.80)
Median (Min, Max)	343.78 (1.67, 1003.33)	566.80 (13.33, 1005.00)
IFN-gamma ELISpot / IL-5 ELISpot ratio		
GMC (95% CI)	4.17 (2.37–7.34)	2.00 (0.75–5.35)
Median (Min, Max)	3.13 (0.50, 391.89)	2.86 (0.13, 14.91)
1 month after the vaccination (D29)	26	9
SARS-CoV-2 DMSO		
IFN-gamma ELISpot		
GMC (95% CI) – SFU/10^6 PBMCs	8.35 (5.38–12.96)	5.82 (2.66–12.72)
Median (Min, Max)	7.46 (0, 41.67)	5.00 (0, 33.33)
IL-5 ELISpot		
GMC (95% CI) – SFU/10^6 PBMCs	5.76 (4.35–7.62)	5.84 (2.92–11.65)
Median (Min, Max)	5.78 (0, 18.33)	5.00 (1.67, 16.67)
IFN-gamma ELISpot / IL-5 ELISpot ratio		
GMC (95% CI)	1.34 (0.74–2.44)	0.80 (0.23–2.74)
Median (Min, Max)	1.13 (0.05, 25.00)	0.60 (0.06, 14.97)
SARS-CoV-2 Peptide 1		
IFN-gamma ELISpot		
GMC (95% CI) – SFU/10^6 PBMCs	51.90 (28.86–93.31)	32.68 (6.80–156.96)
Median (Min, Max)	86.49 (0, 233.33)	68.03 (0, 398.33)
IL-5 ELISpot		
GMC (95% CI) – SFU/10^6 PBMCs	10.29 (7.46–14.21)	9.22 (5.61–15.14)
Median (Min, Max)	12.49 (0, 28.33)	9.97 (0, 21.67)
IFN-gamma ELISpot / IL-5 ELISpot ratio		
GMC (95% CI)	5.24 (2.66–10.33)	3.54 (0.84–15.02)
Median (Min, Max)	5.21 (0.04, 186.67)	3.54 (0.17, 41.67)

	Vaccine group	
	HXP-GPOVac-primed	BNT162b2-primed
SARS-CoV-2 Peptide 2		
IFN-gamma ELISpot		
GMC (95% CI) – SFU/10^6 PBMCs	65.24 (35.28–120.66)	37.25 (8.68–159.81)
Median (Min, Max)	105.64 (0, 385.00)	70.11 (0, 306.67)
IL-5 ELISpot		
GMC (95% CI) – SFU/10^6 PBMCs	11.59 (8.25,16.28)	11.23 (6.76–18.66)
Median (Min, Max)	13.33 (0, 48.33)	13.33 (0, 21.67)
IFN-gamma ELISpot / IL-5 ELISpot ratio		
GMC (95% CI)	5.42 (2.45–11.97)	2.96 (0.63–13.88)
Median (Min, Max)	7.54 (0.03, 181.67)	4.20 (0.05, 36.82)
SARS-CoV-2 PHA		
IFN-gamma ELISpot		
GMC (95% CI) – SFU/10^6 PBMCs	812.41 (393.92–1675.47)	787.52 (152.16–4075.96)
Median (Min, Max)	1472.92 (0, 2316.67)	1571.84 (0, 2230.00)
IL-5 ELISpot		
GMC (95% CI) – SFU/10^6 PBMCs	213.38 (103.16–441.37)	206.21 (57.27–742.54)
Median (Min, Max)	445.86 (0, 1006.67)	354.25 (3.33, 805.00)
IFN-gamma ELISpot / IL-5 ELISpot ratio		
GMC (95% CI)	3.81 (2.28–6.35)	3.41 (1.43–8.12)
Median (Min, Max)	2.87 (0.67, 159.46)	3.44 (0.30, 21.33)
6 months after the vaccination (D169)	26	9
SARS-CoV-2 DMSO		
IFN-gamma ELISpot		
GMC (95% CI) – SFU/10^6 PBMCs	4.72 (3.25–6.86)	3.07 (1.98–4.77)
Median (Min, Max)	2.72 (0, 40.00)	2.72 (0, 13.33)
IL-5 ELISpot		
GMC (95% CI) – SFU/10^6 PBMCs	7.47 (4.18–13.37)	5.62 (2.88–10.96)

	Vaccine group	
	HXP-GPOVac-primed	BNT162b2-primed
Median (Min, Max)	5.78 (0, 941.67)	5.00 (0, 23.33)
IFN-gamma ELISpot / IL-5 ELISpot ratio		
GMC (95% CI)	0.41 (0.21–0.82)	0.31 (0.12–0.84)
Median (Min, Max)	0.57 (0.01, 10.98)	0.30 (0.04, 1.67)
SARS-CoV-2 Peptide 1		
IFN-gamma ELISpot		
GMC (95% CI) – SFU/10^6 PBMCs	12.05 (6.00–24.21)	4.55 (1.68–12.33)
Median (Min, Max)	6.46 (0, 421.67)	2.72 (0, 106.67)
IL-5 ELISpot		
GMC (95% CI) – SFU/10^6 PBMCs	10.96 (6.13–19.59)	7.77 (3.83–15.76)
Median (Min, Max)	8.33 (0, 903.33)	9.97 (0, 23.33)
IFN-gamma ELISpot / IL-5 ELISpot ratio		
GMC (95% CI)	0.81 (0.33–1.96)	0.34 (0.08–1.45)
Median (Min, Max)	1.00 (0.01, 38.73)	0.33 (0.05, 21.33)
SARS-CoV-2 Peptide 2		
IFN-gamma ELISpot		
GMC (95% CI) – SFU/10^6 PBMCs	12.46 (6.34–24.48)	3.43 (1.57–7.52)
Median (Min, Max)	7.46 (0, 233.33)	2.72 (0, 48.33)
IL-5 ELISpot		
GMC (95% CI) – SFU/10^6 PBMCs	10.16 (5.81–17.76)	8.53 (4.16–17.49)
Median (Min, Max)	8.33 (0, 911.67)	15.03 (1.67, 23.33)
IFN-gamma ELISpot / IL-5 ELISpot ratio		
GMC (95% CI)	0.94 (0.44–1.98)	0.23 (0.08–0.64)
Median (Min, Max)	1.00 (0.05, 34.98)	0.20 (0.04 - 3.22)
SARS-CoV-2 PHA		
IFN-gamma ELISpot		
GMC (95% CI) – SFU/10^6 PBMCs	108.19 (34.02–344.00)	24.81 (2.79–220.58)

	Vaccine group	
	HXP-GPOVac-primed	BNT162b2-primed
Median (Min, Max)	439.22 (0, 2060.00)	2.72 (0, 1295.00)
IL-5 ELISpot		
GMC (95% CI) – SFU/10^6 PBMCs	64.52 (26.59–156.54)	32.21 (6.53–158.80)
Median (Min, Max)	80.24 (0, 1091.67)	25.03 (1.67, 615.00)
IFN-gamma ELISpot / IL-5 ELISpot ratio		
GMC (95% CI)	1.49 (0.83–2.69)	0.49 (0.15–1.62)
Median (Min, Max)	1.49 (0.09, 22.89)	0.60 (0.06, 3.82)

Abbreviations: DMSO, dimethyl sulfoxide; ELISpot, enzyme-linked immunospot; PHA, phytohemagglutinin; IFN-γ, interferon-gamma; IL-5, interleukin-5; PBMC, peripheral blood mononuclear cell; SFU, spot-forming units; GMC, geometric mean concentration; CI, confidence interval.
Note: Responses are summarized as GMCs (95% CI) and medians (min, max). IFN-γ and IL-5 expressed as SFU/10^6 PBMCs. IFN-γ/IL-5 ratios summarized as GMCs. DMSO was used as a negative control, whereas PHA was used as a positive control.

Supplementary Table S4 – Incidence density of anti-N seropositivity (anti-N ≥1.1 among baseline anti-N–seronegative participants), symptomatic RT-PCR–confirmed COVID-19 during follow-up and SARS-CoV-2 sequencing results among RT-PCR–confirmed symptomatic COVID-19 cases during follow-up.

**Age group**	**Result**
Overall age (18-75 yrs)	
Anti-N seropositive (S/CO ≥ 1.1) – *n*	48
Person-year	157.21
Incidence rate – per person-year	0.305
Incidence rate – per 100 person-years	30.57
Symptomatic COVID-19 RT-PCR Confirmed – *n*	5
Person-year	197.9
Incidence rate – per person-year	0.03
Incidence rate – per 100 person-years	2.53
Age 18–59 yrs	
Anti-N seropositive (S/CO ≥ 1.1) – *n*	39
Person-year	129.07

Incidence rate – per person-year	0.3
Incidence rate – per 100 person-years	30.22
Symptomatic COVID-19 RT-PCR Confirmed – *n*	3
Person-year	160.4
Incidence rate – per person-year	0.02
Incidence rate – per 100 person-years	1.87
Age 60–75 yrs	
Anti-N seropositive (S/CO ≥ 1.1) – *n*	9
Person-year	28.13
Incidence rate – per person-year	0.32
Incidence rate – per 100 person-years	31.99
Symptomatic COVID-19 RT-PCR Confirmed – *n*	2
Person-year	37.5
Incidence rate – per person-year	0.05
Incidence rate – per 100 person-years	5.33

**Subject ID**	**Study Group**	**Visit**	**Strain**
121011	HXP-GPOVac-primed	Visit 6 (Day 169)	XBB.1.16
122033	HXP-GPOVac-primed	Visit 6 (Day 169)	XBB.1.16
122036	BNT162b2-primed	Visit 6 (Day 169)	XBB.1.5.57
121040	HXP-GPOVac-primed	Visit 5 (Day 85)	unable to perform sequencing
121101	HXP-GPOVac-primed	Visit 6 (Day 169)	unable to perform sequencing

Abbreviations: anti-N, anti–SARS-CoV-2 nucleocapsid immunoglobulin G; RT-PCR, reverse-transcription polymerase chain reaction.

Notes: Events are the number of participants meeting the endpoint during follow-up. Person-years are the total time at risk contributed by participants. Incidence rate = events/person-years; incidence rate per 100 person-years = (events/person-years) × 100. Participants anti-N seropositive at the pre-additional-dose baseline specimen were excluded from the baseline-negative anti-N incidence analysis. Primary-series group indicates the vaccine received in Study 202 (HXP-GPOVac or BNT162b2) prior to the additional HXP-GPOVac dose in Study 203. "Unable to perform sequencing" indicates insufficient sample quantity/quality or failed sequencing.

Supplementary Table S5 – Anti–SARS-CoV-2 nucleocapsid (anti-N) IgG over time after an additional HXP-GPOVac dose, by primary-series group and age stratum (per-protocol population).

	Overall		18 – 59 yrs		≥ 60 yrs	
	HXP-GPOVac-primed (*N* = 167)	BNT162b2-primed (*N* = 52)	HXP-GPOVac-primed (*N* = 135)	BNT162b2-primed (*N* = 42)	HXP-GPOVac-primed (*N* = 32)	BNT162b2-primed (*N* = 10)
Baseline (D1) (Month 6 post-primary; pre-additional dose)						
Anti-N seronegative – *n*	144	48	120	38	24	10
Anti-N IgG GMC – S/CO	0.16	0.18	0.15	0.21	0.2	0.11
(95% CI)	(0.13–0.18)	(0.14–0.24)	(0.12–0.17)	(0.15–0.28)	(0.14–0.30)	(0.05–0.26)
14 days after the vaccination (D15)						
Anti-N – *n*	142	48	118	38	24	10
Anti-N IgG GMC – S/CO	0.16	0.19	0.15	0.22	0.24	0.13
(95% CI)	(0.14–0.19)	(0.15–0.26)	(0.13–0.18)	(0.17–0.29)	(0.16–0.36)	(0.05–0.31)
Anti-N IgG seroresponse – *n* (%)	3 (2.1)	0 (0.0)	2 (1.7)	0 (0.0)	1 (4.2)	0 (0.0)
(95% CI)	(0.4–6.0)	(0.0–7.4)	(0.2–6.0)	(0.0-9.3)	(0.1–21.1)	(0.0–30.8)
Anti-N IgG seropositive – *n* (%)	1 (0.7)	0 (0.0)	0 (0.0)	0 (0.0)	1 (4.2)	0 (0.0)
(95% CI)	(0.0–3.8)	(0.0–7.4)	(0.0–3.0)	(0.0–9.3)	(0.1–21.1)	(0.0–30.8)
p-value	1.000 ‡		-		1.000 ‡	
3 months after the vaccination (D85)						
Anti-N – *n*	139	47	115	37	24	10
Anti-N IgG GMC – S/CO	0.2	0.26	0.19	0.3	0.24	0.17
(95% CI)	(0.16–0.25)	(0.18–0.39)	(0.15–0.24)	(0.19–0.46)	(0.16–0.38)	(0.06–0.52)
Anti-N IgG seroresponse – *n* (%)	16 (11.5)	7 (14.9)	13 (11.3)	5 (13.5)	3 (12.5)	2 (20.0)
(95% CI)	(6.7–18.0)	(6.2–28.3)	(6.2–18.6)	(4.5–28.8)	(2.7–32.4)	(2.5–55.6)
Anti-N IgG seropositive – *n* (%)	13 (9.0)	8 (16.7)	11 (9.2)	7 (18.4)	2 (8.3)	1 (10.0)
(95% CI)	(4.9–14.9)	(7.5–30.2)	(4.7–15.8)	(7.7–34.3)	(1.0–27.0)	(0.3–44.5)
p-value	0.141 †		0.143 ‡		1.000 ‡	
6 months after the vaccination (D169)						

	Overall		18 – 59 yrs		≥ 60 yrs	
	HXP-GPOVac-primed (*N* = 167)	BNT162b2-primed (*N* = 52)	HXP-GPOVac-primed (*N* = 135)	BNT162b2-primed (*N* = 42)	HXP-GPOVac-primed (*N* = 32)	BNT162b2-primed (*N* = 10)
Anti-N – *n*	139	45	115	35	24	10
Anti-N IgG GMC – S/CO	0.21	0.29	0.19	0.32	0.3	0.21
(95% CI)	(0.17–0.26)	(0.19–0.43)	(0.16–0.24)	(0.21–0.48)	(0.19–0.48)	(0.07–0.64)
Anti-N IgG seroresponse – *n* (%)	22 (15.8)	8 (17.8)	17 (14.8)	7 (20.0)	5 (20.8)	1 (10.0)
(95% CI)	(10.2–23.0)	(8.0–32.1)	(8.9–22.6)	(8.4–36.9)	(7.1–42.2)	(0.3–44.5)
Anti-N IgG seropositive – *n* (%)	19 (13.2)	12 (25.0)	16 (13.3)	10 (26.3)	3 (12.5)	2 (20.0)
(95% CI)	(8.1–19.8)	(13.6–39.6)	(7.8–20.7)	(13.4–43.1)	(2.7–32.4)	(2.5–55.6)
p-value	0.054 †		0.059 †		0.618 ‡	
12 months after the vaccination (D337)						
Anti-N – *n*	137	46	113	36	24	10
Anti-N IgG GMC – S/CO	0.25	0.32	0.22	0.39	0.41	0.16
(95% CI)	(0.20–0.31)	(0.23–0.45)	(0.17–0.28)	(0.27–0.56)	(0.23–0.74)	(0.07–0.36)
Anti-N IgG seroresponse – *n* (%)	30 (21.9)	8 (17.4)	23 (20.4)	8 (22.2)	7 (29.2)	0 (0.0)
(95% CI)	(15.3–29.8)	(7.8–31.4)	(13.4–29.0)	(10.1–39.2)	(12.6–51.1)	(0.0–30.8)
Anti-N IgG seropositive – *n* (%)	32 (22.2)	16 (33.3)	25 (20.8)	14 (36.8)	7 (29.2)	2 (20.0)
(95% CI)	(15.7–29.9)	(20.4–48.4)	(14.0–29.2)	(21.8–54.0)	(12.6–51.1)	(2.5–55.6)
p-value	0.123 †		0.046 †*		0.692 ‡	

Abbreviations: anti-N, anti–SARS-CoV-2 nucleocapsid immunoglobulin G; CI, confidence interval; GMC, geometric mean concentration; PP, per-protocol; S/CO, signal-to-cutoff.

Note: Baseline anti-N IgG was measured using the pre-vaccination serum specimen collected at the Month 6 visit in Study 202 (closest available pre-dose sample) and used as the baseline specimen for Study 203 anti-N analyses. This table includes only participants with available baseline anti-N IgG results who were seronegative at baseline and had paired results. Two-sided p-values are as †overall p-value (2-sided) based on Chi-square test or ‡overall p-value (2-sided) based on Fisher's exact test, * indicates statistical significance (p-value < 0.05). Seroresponse refers to having ≥ 4-fold rise in Anti-N IgG GMC (S/CO) from baseline, whereas seropositive refers to having Anti-N IgG GMC (S/CO) ≥ 1.1 among seronegative participants.

Supplementary Table S6 – Severity grading scale for solicited adverse events.

Adverse Event	Mild (Grade 1)	Moderate (Grade 2)	Severe (Grade 3)	Potentially Life-Threatening (Grade 4)
Systemic Illness				
Illness or clinical AE (as defined according to applicable regulations)	No or minimal interference with usual activities; no medical intervention/ therapy required	Marked limitation in ability to perform usual activities; medical intervention/ therapy required	Marked limitation in ability to perform usual activities; medical intervention/ therapy required	Inability to perform basic functions OR Medical or operative intervention indicated to prevent permanent impairment, persistent disability, or death
Local Reaction to Injectable Product				
Injection site pain (pain without touching) OR Tenderness (pain when area touched)	Pain/tenderness causing no or minimal limitation of use of limb	Pain or tenderness causing greater than minimal limitation of use of limb	Pain/tenderness causing inability to perform usual activities	Pain/tenderness causing inability to perform basic functions OR Hospitalization indicated
Injection site erythema or induration	2.5 to < 5 cm in diameter OR 6.25 to < 25 cm2 surface area	≥ 5 to < 10 cm in diameter OR ≥ 25 to < 100 cm2 surface area	≥ 10 cm in diameter OR ≥ 100 cm2 surface area OR Ulceration OR Secondary infection OR Phlebitis OR Sterile abscess OR Drainage	Potentially life-threatening consequences (e.g., abscess, exfoliative dermatitis, necrosis involving dermis or deeper tissue)
Injection site pruritus	Itching localized to the injection site that is relieved spontaneously or in < 48 hours of treatment	Itching beyond the injection site that is not generalized OR Itching localized to the injection site requiring ≥ 48 hours treatment	Generalized itching causing inability to perform usual social & functional activities	N/A
Systemic (General)				
Acute systemic allergic reaction	Localized urticaria (wheals) with no medical intervention indicated	Localized urticaria with medical intervention indicated OR Mild angioedema with no	Generalized urticaria OR Angioedema with medical intervention indicated OR	Acute anaphylaxis OR Life-threatening bronchospasm OR laryngeal edema

Adverse Event	Mild (Grade 1)	Moderate (Grade 2)	Severe (Grade 3)	Potentially Life-Threatening (Grade 4)
		medical intervention indicated	Symptomatic mild bronchospasm	
Fever – °C	38.0 – 38.6	38.7 – 39.3	39.4 – 40.5	> 40.5
Myalgia (generalized)	Muscle pain causing no or minimal interference with usual social and functional activities	Muscle pain causing greater than minimal interference with usual social and functional activities	Muscle pain causing inability to perform usual social and functional activities	Disabling muscle pain causing inability to perform basic self-care functions
Headache	Symptoms causing no or minimal interference with usual social and functional activities	Symptoms causing greater than minimal interference with usual social and functional activities	Symptoms causing inability to perform usual social and functional activities	Symptoms causing inability to perform basic self-care functions OR Hospitalization indicated OR Headache with significant impairment of alertness or other neurologic function
Chills	Symptoms causing no or minimal interference with usual social and functional activities	Symptoms causing greater than minimal interference with usual social and functional activities	Symptoms causing inability to perform usual social and functional activities	N/A
Fatigue	Symptoms causing no or minimal interference with usual social and functional activities	Symptoms causing greater than minimal interference with usual social and functional activities	Symptoms causing inability to perform usual social and functional activities	Incapacitating symptoms of fatigue or malaise causing inability to perform basic self- care functions
Malaise	No interference with activity	Some interference with activity	Prevents daily activity	Hospitalization
Arthralgia	No interference with activity	Some interference with activity	Prevents daily activity	Hospitalization
Nausea/Vomiting	No interference with activity	Some interference with activity	Prevents daily activity	Hospitalization

Adverse Event	Mild (Grade 1)	Moderate (Grade 2)	Severe (Grade 3)	Potentially Life-Threatening (Grade 4)
Tachycardia – beats per minute	101 – 115	116 – 130	> 130	ER visit or hospitalization for arrhythmia
Bradycardia – beats per minute	50 – 54	45 – 49	< 45	ER visit or hospitalization for arrhythmia
Respiratory Rate – breaths per minute	17 – 20	21 – 25	> 25	Intubation
